# Oxidative stress genes define two subtypes of triple-negative breast cancer with prognostic and therapeutic implications

**DOI:** 10.3389/fgene.2023.1230911

**Published:** 2023-07-13

**Authors:** Shenting Liu, He Xu, Ying Feng, Ulf D. Kahlert, Renfei Du, Luz Angela Torres-de la Roche, Kai Xu, Wenjie Shi, Fanshuai Meng

**Affiliations:** ^1^ Department of Oncology Medicine, Hainan Cancer Hospital, Haikou, Hainan, China; ^2^ Department of Thyroid and Breast Surgery, Xuzhou Municipal Hospital Affiliated to Xuzhou Medical University, Xuzhou, China; ^3^ Molecular and Experimental Surgery, University Clinic for General- Visceral- Vascular- and Trans-Plantation Surgery, Medical Faculty University Hospital Magdeburg, Otto-von Guericke University Magdeburg, Germany; ^4^ University Hospital for Gynecology, Pius-Hospital, University Medicine Oldenburg, Carl von Ossietzky University Oldenburg, Oldenburg, Germany; ^5^ Translational and Trauma Surgery Laboratory, University of Ulm, Ulm, Germany

**Keywords:** triple-negative breast cancer, oxidative stress, molecular subtype, tumor microenvironment, immunotherapy, relapse-free survival

## Abstract

**Introduction:** Oxidative stress (OS)-related genes have been confirmed to be closely related to the prognosis of triple-negative breast cancer (TNBC) patients; despite this fact, there is still a lack of TNBC subtype strategies based on this gene guidance. Here, we aimed to explore OS-related subtypes and their prognostic value in TNBC.

**Methods:** Data from The Cancer Genome Atlas (TCGA)-TNBC and Sequence Read Archive (SRA) (SRR8518252) databases were collected, removing batch effects using a combat method before analysis. Consensus clustering analysis identified two OS subtypes (clusters A and B), with cluster A showing a better prognosis. Immune infiltration characteristics were analyzed using ESTIMATE and single-sample gene set enrichment analysis (ssGSEA) algorithms, revealing higher ImmuneScore and ESTIMATEscore in cluster A. Tumor-suppressive immune cells, human leukocyte antigen (HLA) genes, and three immune inhibitors were more prevalent in cluster A.

**Results:** An eight-gene signature, derived from differentially expressed genes, was developed and validated as an independent risk factor for TNBC. A nomogram combining the risk score and clinical variables accurately predicted patient outcomes. Finally, we also validated the classification effect of subtypes using hub markers of each subtype in the test dataset.

**Conclusion:** Our study reveals distinct molecular clusters based on OS-related genes to better clarify the reactive oxygen species (ROS)-mediated progression and the crosstalk between the ROS and tumor microenvironment (TME) in this heterogenetic disease, and construct a risk prognostic model which could provide more support for clinical treatment decisions.

## 1 Introduction

According to the data from the Global Burden of Disease study, breast cancer (BC) accounts for 30% of all female cancers, and its incidence rate is still increasing since the past decade (Chen et al., 2020). In 2022, an estimate of 259,827 new cases and 124,002 deaths from breast cancer was observed in the United States, contributing to this disease as the cancer having the second highest mortality rate after lung cancer worldwide (Xia et al., 2022). According to the expression of molecular markers on the surface of breast cancer cells, breast cancer can be divided into hormone receptor (HR)-positive, human epidermal growth factor receptor 2 (HER2)-enriched, and triple-negative breast cancer (TNBC) subtypes (absence of estrogen receptor (ER), progesterone receptor, and HER2 expression). Among these subtypes, TNBC accounts for 10%–15% of all BC cases with a high recurrence rate (Brewster et al., 2014). Although many scholars have made efforts to explore tumor biology and develop new treatment for this subtype, the achievements are still unsatisfactory with an extremely poor prognosis with a median overall survival of 10–13 months in metastatic BC patients (Shi et al., 2019; Waks and Winer, 2019). Given the heterogeneity of TNBC patients, exploring new targets that can precisely predict the prognosis and can be treated as biomarkers is needed.

Oxidative stress (OS) is the imbalance of oxidation and antioxidant systems in the body, resulting in the production of excessive peroxides and free radicals, which leads to the occurrence of various diseases and tumors via damage to the proteins, lipids, and DNA of cells (Forman and Zhang, 2021; Pisoschi et al., 2021). Reactive oxygen species (ROS) refers to free radicals and non-radicals derived from oxygen; they are produced by the mitochondrial respiratory chain (Jones, 2008). The physiological concentration of ROS is crucial to maintaining cell survival, while the OS will occur when the production of ROS exceeds the individual degradation capacity. ROS can also cause DNA damage, thereby leading to genetic instability and tumorigenesis (Liu Y. et al., 2022). Excessive ROS has been reported to be involved in the pathological and progressive process of hypertension, Alzheimer’s disease, gastric cancer, myeloid leukemia, and breast cancer (Gella and Durany, 2009; Harrison and Gongora, 2009; Mazzuferi et al., 2021; Trombetti et al., 2021; Wu et al., 2021). In the mitochondrial respiratory chain, ROS-mediated oxidative stress can significantly damage mitochondrial DNA (mtDNA) and enhance the incidence of new mutations, and the accumulation of such new mtDNA mutations can increase the incidence of BC (Chen et al., 2022). In addition, the role of the tumor microenvironment (TME) in the development of various tumors has recently been recognized (Bejarano et al., 2021); different cell types and soluble factors in the TME can promote the progression and metastasis of TNBC, and hypoxia is an important factor in promoting this tumor biology process (Deepak et al., 2020; Lei et al., 2021). Although some previous studies have confirmed the function of OS in the tumorigenesis and aggressiveness of BC, little was known about ROS-mediated progression in the TME of TNBC (Brown and Bicknell, 2001; Wilkerson and Hayes, 2010; Nourazarian et al., 2014; Wu et al., 2021).

In the aforementioned concepts, we aim to establish different molecular subtypes in TNBC based on OS-related genes to better clarify the ROS-mediated progression and the crosstalk between the ROS and TME in this heterogenetic disease, and construct a risk prognostic model which could provide more support for clinical treatment decisions.

## 2 Material and methods

### 2.1 Data collection

Transcriptome RNA sequencing (RNA-seq) data and corresponding clinical data on TNBC were downloaded from two public databases, namely, The Cancer Genome Atlas (TCGA) (https://portal.gdc.cancer.gov/) (TCGA-TNBC) (including 188 TNBC samples) and the Sequence Read Archive (SRA) databases (https://www.ncbi.nlm.nih.gov/sra) with code SRR8518252 (including 360 TNBC samples). The raw RNA-seq data from TCGA database were processed using R software, and raw data from the SRA database were downloaded using the SRA Toolkit. Meanwhile, the patients without follow-up information and complete relapse-free survival (RFS) outcomes were excluded from our study; so, a total of 548 TNBC patients were included in the current study. All TNBC samples included in this study were used as the training set, and 40% of TNBC samples were randomly selected as the internal validation dataset (testing dataset). Lastly, we also extracted 1,399 oxidative stress proteins from the GeneCards database (https://www.genecards.org/) based on a previous study.

### 2.2 Batch effect correction

After the aforementioned transcriptome sequencing raw expression data from different platforms were combined and log2-transformed, the expression profiles of OS-related genes were extracted from the normalized matrix based on the names of the obtained 1,399 OS-related genes, all of which were implemented using the R package. To eliminate batch differences between two datasets, we applied the ComBat method from the R package “sva” for standardization. In addition, principal component analysis (PCA) was conducted to evaluate the performance of batch effect removal.

### 2.3 Screening of OS-associated prognostic genes

After correcting for batch effect, we selected the 1,399 OS gene expression profiles from the combined matrix. Then, univariate Cox regression analysis was performed to evaluate the relapse-free survival (RFS)-related genes. The screening criteria were set as a *p*-value less than 0.05, and the top 10 results of the analysis were visualized on a forest map.

### 2.4 Identification of OS subtypes by consensus clustering

Based on the OS-related prognostic genes, unsupervised consensus clustering analysis was conducted to identify OS molecular subtypes and define the number of clusters via the “ConsensusClusterPlus” R package in 548 TNBC samples. To ensure the stratification stability, after 1,000 iterations of parameters, the optimal K value was determined, which represents the optimal number of clusters out of 2–9 clusters. For showing the distribution difference of OS subtypes, we carried out principal component analysis. We also compared the difference in the RFS between different clusters in the total dataset to evaluate the clinical value of OS subtypes, which were analyzed using the log-rank test. The relationship between subtypes and clinical factors was also analyzed.

### 2.5 Immune landscape of OS subtypes

To explore the immune infiltration characteristics of the OS clusters, here, the ESTIMATE algorithm and “Estimate” R package were used for calculating the proportion of immune matrix components in different OS molecular subtypes, including the ImmuneScore, StromalScore, and ESTIMATEScore. The enrichment level of tumor-infiltrating immune cells (TICs) in the TME between the OS subtypes was compared using a single-sample gene set enrichment analysis (ssGSEA) program. Meanwhile, we also assessed the differential expression of the human leukocyte antigen (HLA) genes between different OS subtypes. Lastly, to estimate the associations between the OS subtypes and the efficacy of immune checkpoint blockade (ICB) therapy, we analyzed the differential expression levels of three common immune inhibitors in different subtypes. A *p*-value of less than 0.05 represented the significant difference.

### 2.6 Differentially expressed gene screening

We use prediction analysis for microarray (PAM) to validate the subtypes. Differentially expressed genes (DEGs) of each subtype were analyzed using the “limma” package. The filter criteria were set to an adjusted *p*-value < 0.001. The result of the analysis was visualized in a volcano map utilizing the “ggplot2” package. In addition, we extracted the DEGs between different subtypes for further analysis.

### 2.7 Construction of the OS-related signature

In the training set, univariate Cox regression analysis was applied for determining the OS-related DEGs associated with the RFS of TNBC patients. Next, these genes were analyzed using a machine learning method, called least absolute shrinkage and selection operator (LASSO), to optimize the number of genes. Then, we attempted to develop an optimal prognostic signature based on the coefficient (Coef) from multivariate Cox regression analysis. The model was constructed using the following formula: risk score = expression of gene 1 × Coef + expression of gene 2 × Coef +…+ expression of gene n × Coef. For validating the predictive power of the OS-related risk signature, we compared the survival status between high- and low-risk groups with the log-rank test and drew the time-dependent receiver operating characteristic (tROC) curve in the training and testing datasets, which were conducted using the R package survminer. To further ascertain the clinical value of the OS-related risk signature 161, we compared it with other clinical parameters. This was assessed using Cox regression analyses.

### 2.8 Nomogram based on the signature model and function enrichment

Nomograms are a visualization tool widely used to assess the disease prognosis or other clinical outcomes in cancer patients. So based on significant clinical variables and the risk score of the signature, we established a nomogram in the training set to predict the probability of 1-, 3-, and 5-year RFS for patients with TNBC. Then, to validate the reliability of the nomogram between the predicted RFS and actual RFS rates, a calibration curve was drawn using the bootstrap method (1,000 replicates). In addition, to assess the predictive accuracy of the nomogram, we calculated the concordance index (C-index) of the model.

In this study, Gene Ontology (GO) functional and Kyoto Encyclopedia of Genes and Genomes (KEGG) pathway enrichment analyses were conducted using the “clusterProfiler” package, which aims to explore the candidate mechanism of the DEGs in different OS subtypes. GO and the KEGG terms with a q-value of less than 0.05 were defined as significantly enriched. Additionally, the top five results of the enrichment analysis were shown in Circos diagrams.

### 2.9 Identifying and validating hub genes of each subtype

Considering the clinical application, using too many genes to assist in clinical decisions is not possible; so we use prediction analysis for microarray (PAM) algorithms to select hub genes of each subtype, and the standard is keeping only features with a positive PAM score in one subtype. To test the robustness of the subtype, we randomly sampled 40% of the original dataset for subtype prediction and performed survival analysis using the nearest template prediction (NTP) algorithms and KM analysis, respectively.

## 3 Results

### 3.1 Data processing

**FIGURE 1:**
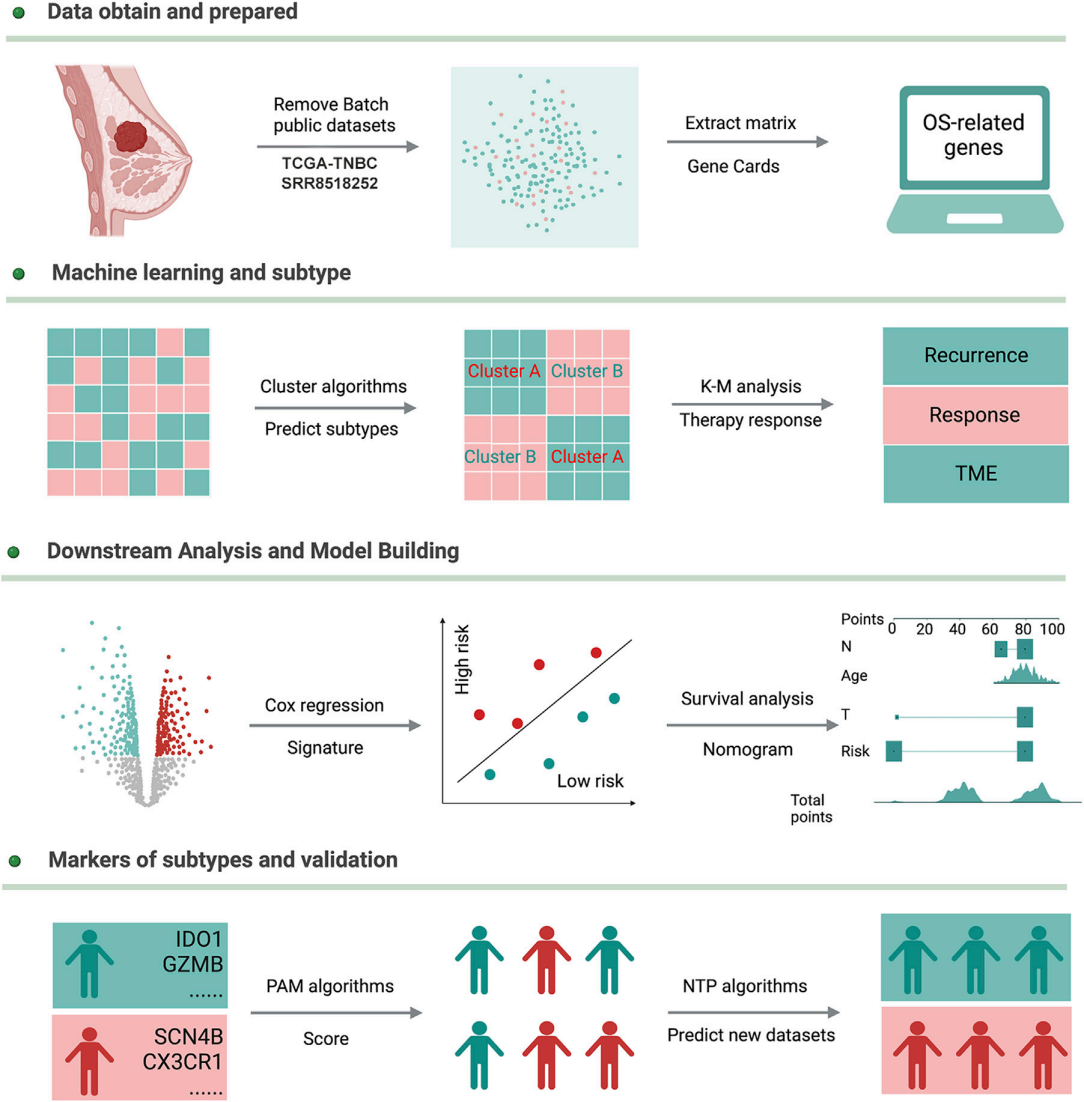
Workflow of this study.

**FIGURE 2:**
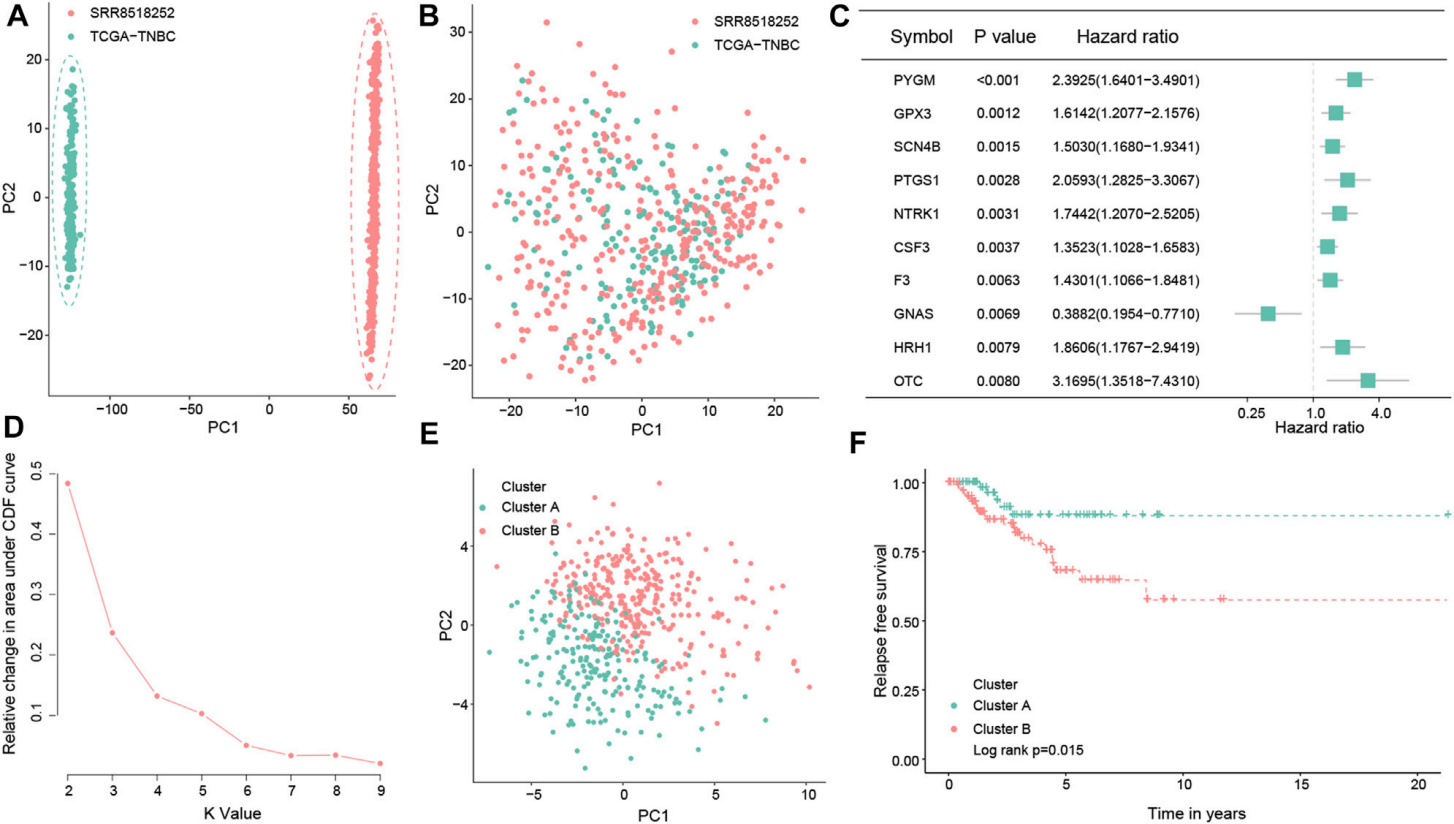
Consensus clustering of the molecular subtype based on the OS-related prognostic genes in TNBC. Principal component analysis for the two TNBC datasets before **(A)** and after merging **(B)**. Forest map showing the top 10 OS-related genes correlated with the RFS in the total dataset (*p* < 0.05) **(C)**. Determination of the optimal cluster number, and *k* = 2 was considered to be the best clustering number **(D)**. Principal component analysis of the OS-related prognostic genes to distinguish cluster A from cluster B samples in TNBC **(E)**. Kaplan–Meier survival analysis between the two OS clusters in the total dataset **(F)**.

The flowchart of this study is shown in Figure 1. Enrolled 548 TNBC samples were obtained from TCGA and SRA databases, respectively. The detailed clinical information on patients is given in Supplementary Table S1. We used the ComBat method to correct batch effects after combining OS-related gene expression data from the two different datasets. Before the transformation of independent datasets, there was an obvious batch effect by the PCA results (Figure 2A). However, two datasets clustered together after the transformation (Figure 2B), demonstrating that we successfully removed the batch effect in cross-platform normalization.

### 3.2 Identification of oxidative stress subtypes in TNBC

By conducting univariate Cox regression analysis in the total dataset, we ultimately identified 61 OS-related genes correlated with the RFS of TNBC patients (*p* < 0.05; Figure 2C; Supplementary Figure S1) for the subsequent determination of the OS subtypes of TNBC. According to the expression levels of the aforementioned 61 prognostic genes, two different OS-related clusters were determined by consistent cluster analysis (cluster A vs. cluster B) (Figure 2D; Supplementary Figure S2). At the same time, the PCA results supported the stratification when *k* = 2, that is, the OS-related prognostic genes successfully distinguished cluster A from cluster B samples in TNBC (Figure 2E). Moreover, the log-rank test indicated that TNBC patients in cluster A had a better prognosis than those in cluster B (***p* = 0.015;** Figure 2F).

### 3.3 Subtype with the clinical factor

**FIGURE 3:**
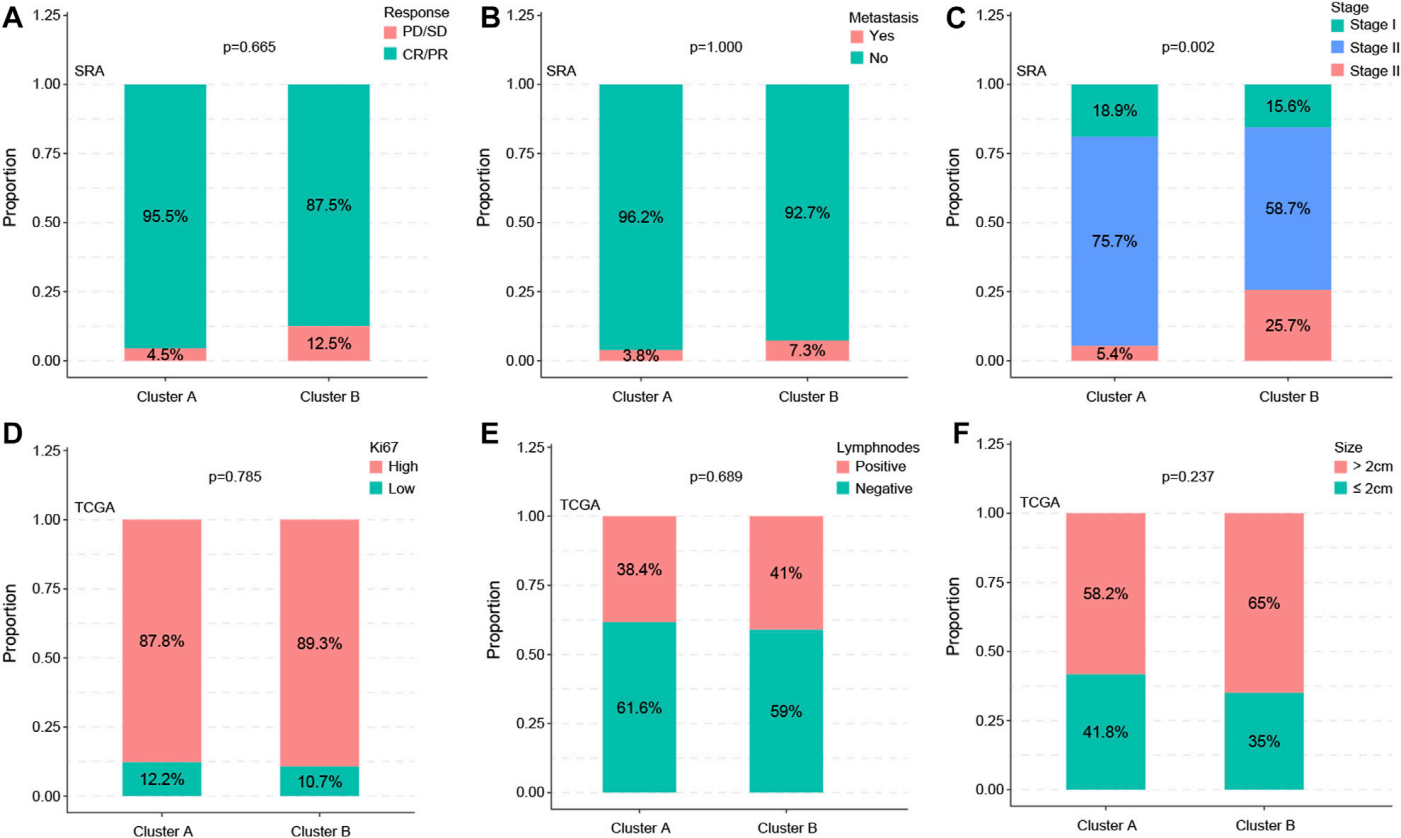
Subtypes are associated with clinic factors. The distribution of the metastasis and therapy response of the two variables showed no significance between subtypes (*p* = 0.665 vs. *p* = 1.000) **(A,B)**, whereas the stage showed a significant difference (*p* = 0.002) **(C)**. Statistical results of KI67, lymph node status, and statistical distribution of the tumor size were also insignificant (*p* = 0.665 vs. *p* = 0.689 vs. *p* = 0.237) **(D–F)**.

By analyzing the correlations between subtypes and clinical variables, we found that the distribution of the two variables metastasis and therapy response was not significant between subtypes (*p* = 0.665 vs. *p* = 1.000) (Figures 3A, B), whereas the stage showed a significant difference (*p* = 0.002) (Figure 3C). The statistical results of KI67, lymph node status, and statistical distribution of the tumor size were also insignificant (*p* = 0.665 vs. *p* = 0.689 vs. *p* = 0.237) (Figures 3D–F).

### 3.4 The immune landscape of oxidative stress subtypes

**FIGURE 4:**
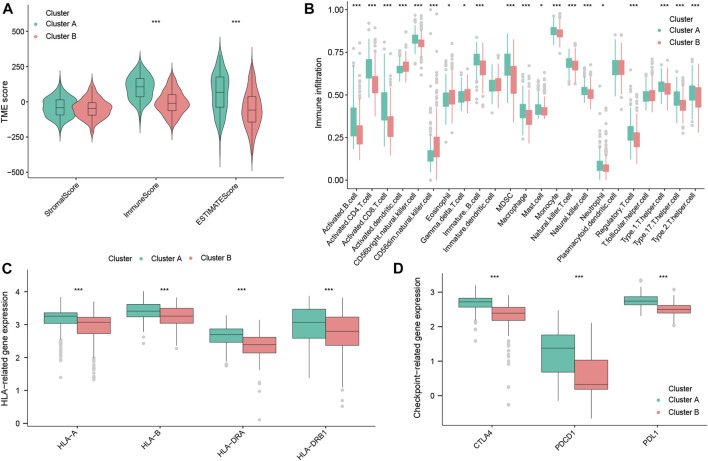
Immune landscape of OS subtypes in the total datasets. StromalScore, ImmuneScore, and ESTIMATEscore between the two OS subtypes **(A)**. Abundance of TICs between the two OS subtypes **(B)**. Expression of HLA genes between the two OS subtypes **(C)**. Expression of three common immune checkpoint molecules between the two OS subtypes **(D)**.

To further explain the mechanisms underlying the differences in the prognosis between the two OS subtypes, we explored the composition of TICs, the HLA, and immune checkpoints in these two subtypes. First, the ImmuneScore and ESTIMATEscores of TNBC patients with cluster A were both higher than those of cluster B (Figure 4A), suggesting that there is a difference in the tumor microenvironment between the two subtypes. By ssGSEA analysis, we obtained the infiltration level of tumor-infiltrating immune cells in the two subtypes. We found that tumor-suppressive immune cells were more infiltrated in cluster A (such as activated B cells, activated CD8^+^ T cells, and natural killer (NK) cells). Interestingly, we also found that tumor-promoting immune cells, regulatory T cells (Tregs), appeared highly infiltrated in cluster A (Figure 4B). This would reveal that cluster A could be an immune-activated subtype, while cluster B could be an immunosuppressive subtype. Moreover, as shown in Figure 4C, cluster A had high expression levels of HLA-A, HLA-B, HLA-DRA, and HLA-DRB1 (*p* < 0.001). Notably, in the analysis of three common immune checkpoints between the two subtypes, our results showed that the expression of PDCD1 (also known as PD-1), CTLA-4, and PDL1 was all significantly increased in the cluster A subtype (*p* < 0.001; Figure 4D). Summing up, the aforementioned results indicated that cluster A may be a tumor-suppressive subtype. At the same time, TNBC patients with cluster A may be more sensitive to ICB therapy, as well as being a target population.

### 3.5 Construction of the OS-related gene prognostic signature

**FIGURE 5:**
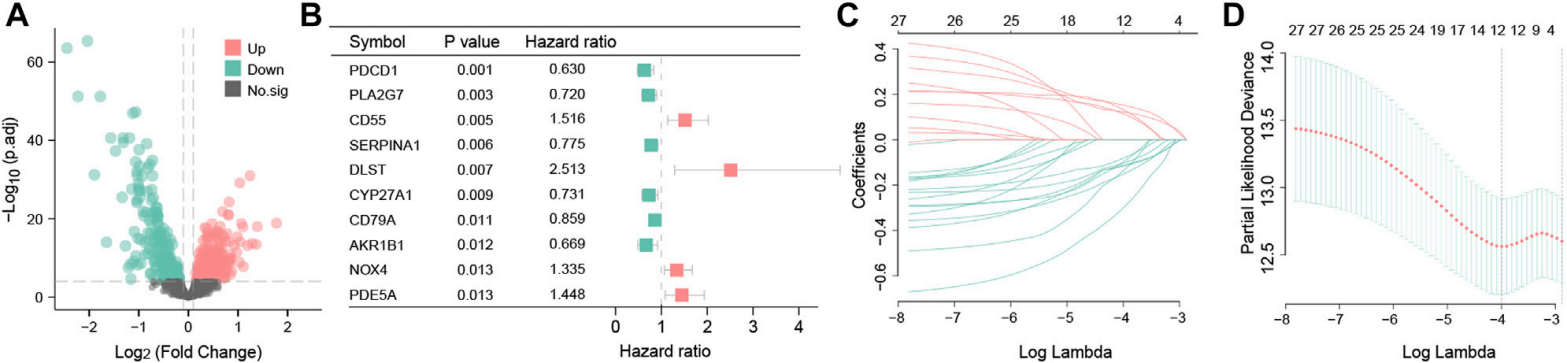
Differential expression analysis and construction of the OS-related prognosis signature. Volcano map of 441 OS-related DEGs between clusters A and B. Red represents the upregulated genes; green represents the downregulated genes; and black represents genes with no statistical difference **(A)**. Forest plot showing the top 10 OS-related genes correlated with RFS among 441 DEGs (*p* < 0.05) **(B)**. The partial likelihood deviance plot is based on the LASSO regression model in the 10-fold cross-validation **(C)**. LASSO coefficient profiles of the 12 OS-associated genes are determined by the optimal lambda **(D)**.

Having the filter criteria of q-value < 0.001, a total of 441 OS-related DEGs were screened from clusters A and B, and among them, 232 were upregulated and 209 were downregulated genes (Figure 5A; Supplementary Table S2). These 441 OS-related DEGs were utilized to build the prognostic signature. First, 27 candidate OS-related genes correlated with the RFS in TNBC patients were selected using univariate Cox regression (Figure 5B; Supplementary Table S3). Next, LASSO regression analysis picked out 12 optimal OS-related prognostic genes with non-zero coefficients (Figures 5C, D). Subsequently, through multivariate Cox regression analysis, eight OS-related genes (*PDCD1*, *CSF2*, *IL6*, *AGTR1*, *SERPINA1*, *CYP27A1*, *GCLC*, and *KNG1*) were associated with the RFS of TNBC patients (*p* < 0.05). Based on the regression coefficients, an eight-gene signature was constructed (Supplementary Table S4). The risk score of the signature was as follows: risk score = (−0.329525354) × PDCD1 + (−0.316236293) × CSF2 + (0.16509038) × IL6 + (0.232422544) ×AGTR1 + (−0.220580168) × SERPINA1 + (−0.285188297) × CYP27A1 + (−0.697833762) × GCLC + (−0.348399969) × KNG1.

### 3.6 Evaluation and validation of OS-related gene signature efficacy

**FIGURE 6:**
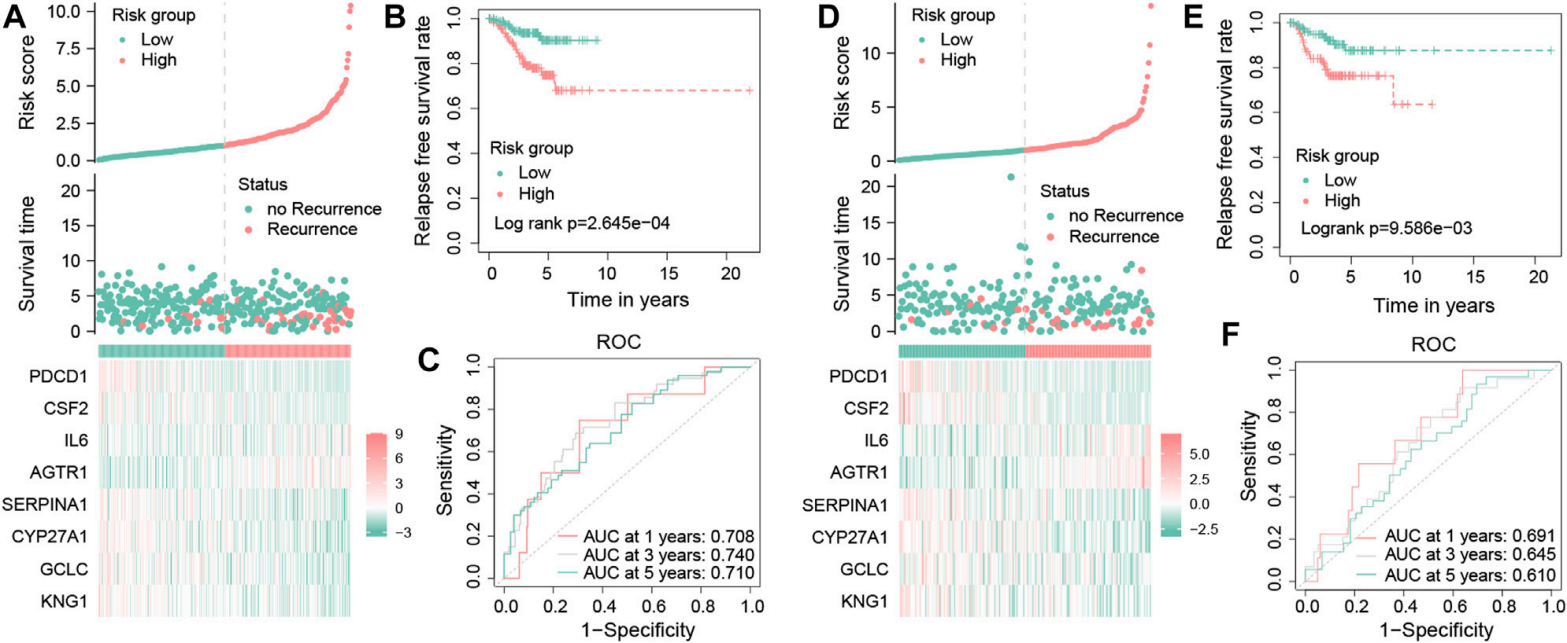
Validation of the OS-related prognostic signature in TNBC patients. Risk score distribution, survival status distribution, and the expression of eight OS-related genes in low- and high-risk groups from the training dataset **(A)**. Kaplan–Meier survival analysis of RFS between the low- and high-risk groups from the training dataset **(B)**. Time-dependent ROC curves of 1-, 3-, and 5-year RFS predicted by the prognostic signature from the training dataset **(C)**. Risk score distribution, survival status distribution, and the expression profile of eight OS-related genes in low- and high-risk groups from the testing dataset **(D)**. Kaplan–Meier survival analysis of RFS between the low- and high-risk groups from the testing dataset **(E)**. Time-dependent ROC curves of 1-, 3-, and 5-year RFS predicted by the prognostic signature from the testing dataset **(F)**.

The risk scores of each TNBC patient will be obtained using the aforementioned formula; patients will be divided into two different groups, one for a high-risk group with a risk score more than the median value and another for the low-risk group. In the training dataset, these results showed that the mortality occurrence depended on the risk score (Figure 6A). Moreover, as shown in the KM survival curve, low-risk patients had significantly longer relapse-free survival time than high-risk patients (***p* = 2.645e−04;** Figure 6B). In addition, the area under the curve (AUC) values of the tROC curves for predicting the RFS were 0.708, 0.740, and 0.710 (Figure 6C), suggesting that the prognostic risk signature for TNBC has favorable predictive sensitivity. The testing dataset was used to validate the predictive performance of the model, and we showed the risk distribution of the model and gene expression in the samples (Figure 6D). Then, we found that the conclusion of the RFS between groups was also consistent (log-rank test, *p* = 9.586e−03) (Figure 6E). Similarly, the 1-, 3-, and 5-year tROC curves further supported the aforementioned conclusion that our signature had good predictive efficacy for RFS outcomes of TNBC patients (AUC = 0.691, 0.645, and 0.610, respectively) (Figure 6F). In general, the aforementioned results proved that this prognostic risk model was considered reliable.

### 3.7 Clinical value of the OS-related gene signature

**FIGURE 7:**
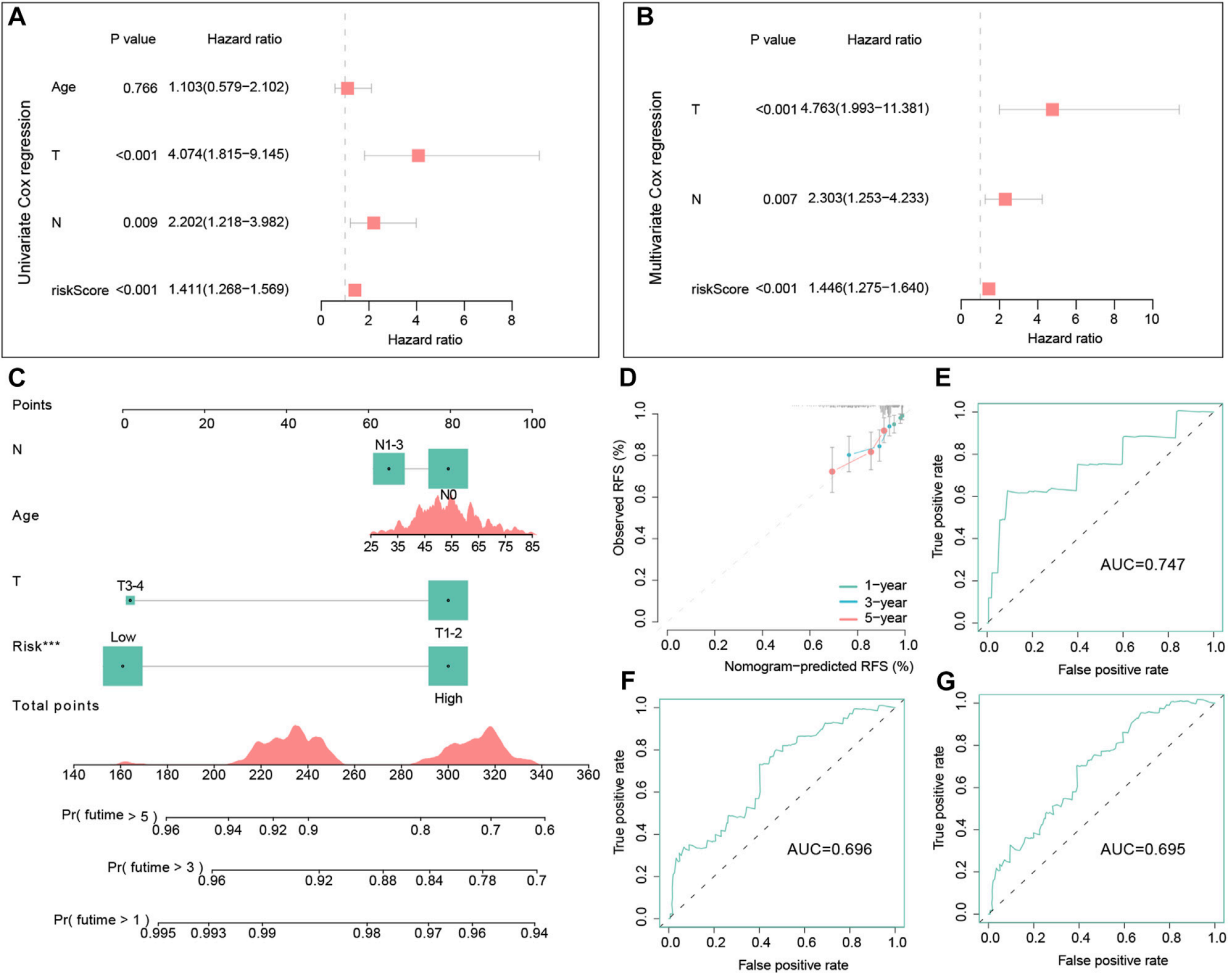
Independent prognostic analysis of the signature and the construction and evaluation of the nomogram in the training dataset. Univariate Cox regression analysis of the signature and other clinical factors in the training set **(A)**. Multivariate Cox regression analysis of the signature and other clinical factors in the training set **(B)**. The nomogram consists of the age, T stage, N stage, and risk score to predict the probability of 1-, 3-, and 5-year RFS in TNBC patients **(C)**. Calibration curves of 1-, 3- and 5-year RFS in TNBC patients were predicted by the nomogram **(D)**. Time-dependent ROC curves of 1-, 3-, and 5-year RFS predicted by the nomogram **(E–G)**.

Cox regression analyses in the training dataset were conducted to assess the clinical value of the signature. In the univariate Cox regression model, the hazard ratio (HR) was 1.411 with 95% confidence interval (CI) ranging from 1.268 to 1.569 (*p* < 0.001) (Figure 7A). This conclusion suggested that the signature was a risk factor for the RFS of TNBC patients. Meanwhile, multivariate Cox regression also demonstrated that this signature was also an independent risk factor for TNBC patients compared with other clinic characteristics (HR = 1.446, 95% CI =1.275–1.640, and *p* < 0.001) (Figure 7B).

### 3.8 Nomogram predicting RFS

In the training set, we successfully built a visualized nomogram combined with several significant clinical factors to predict RFS probability (Figure 7C). The C-index of this model was 0.725, which showed a good capacity in predicting the RFS for TNBC patients. Then, calibration analysis demonstrated that this nomogram had high accuracy because the predicted curve was close to the ideal curve (Figure 7D). Lastly, the tROC curves of this nomogram also illustrated good accuracy for predicting patient outcomes (AUC = 0.747, 0.696, and 0.695, respectively) (Figures 7E–G).

### 3.9 Different biological functions of subtypes

In terms of the potential biological functions of the DEGs, we found that these DEGs were mainly involved in the response to oxidative stress (Supplementary Figure S3A). The important pathways for KEGG enrichment were mitogen-activated protein kinase (MAPK) and tumor necrosis factor (TNF) signaling pathways (Supplementary Figure S3B).

## 4 Subtypes could be predicted by core genes

**FIGURE 8:**
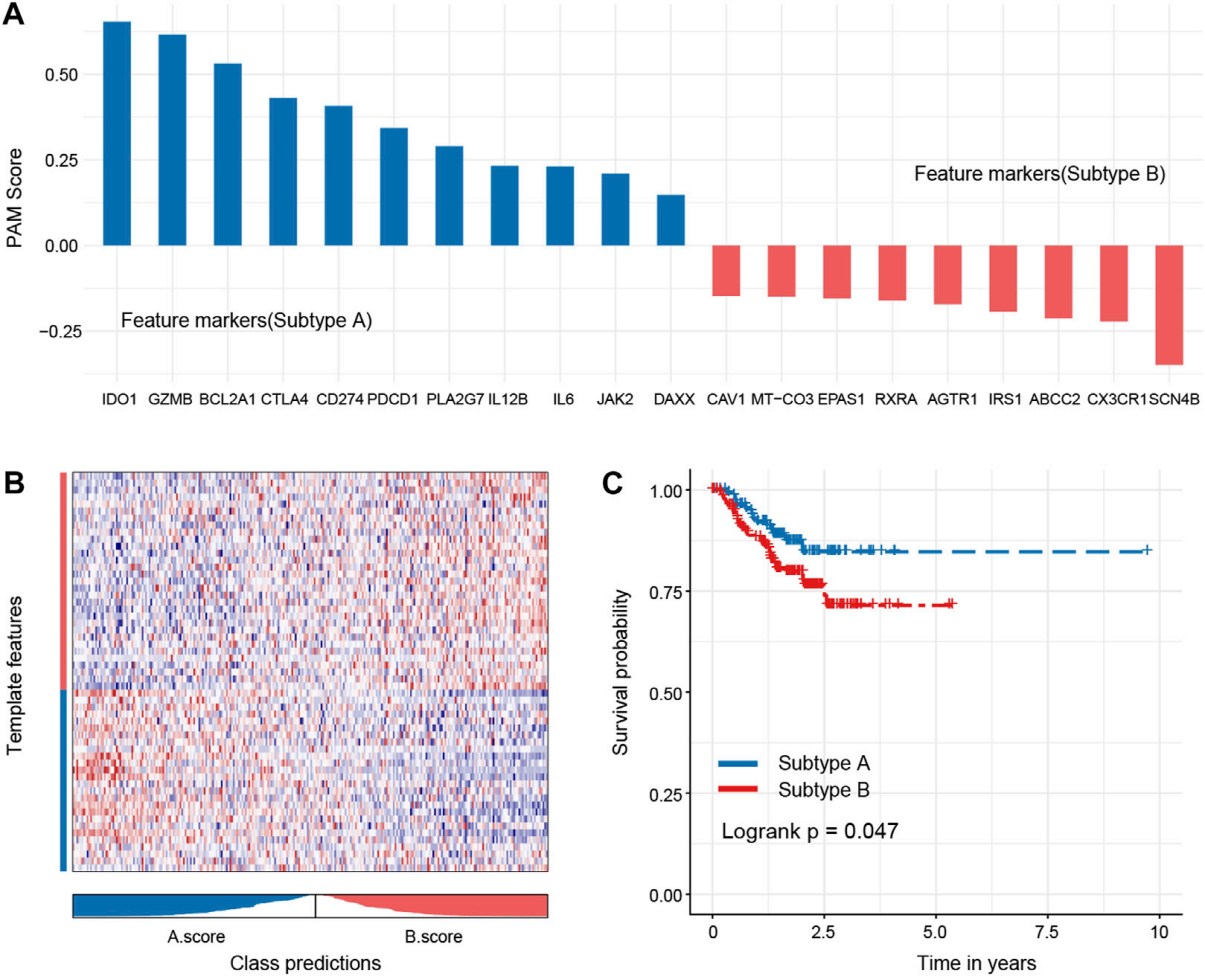
Hub genes of each subtype and robustness of test subtypes in a random dataset. Eleven hub genes of subtype A and 10 hub genes of subtype B were identified **(A),** and these genes could better distinguish the oxidized subtypes of TNBC **(B)**. The subtype A shows a better outcome than the subtype B **(C)**.

To test the robustness of the subtype, we randomly sampled 40% of the original dataset for subtype prediction and performed survival analysis. The analysis results suggested that the core genes could better distinguish the oxidized subtypes of TNBC and further confirmed that the recurrence-free survival time of the A subtype was longer than that of the B subtype, and there was statistical significance between the groups (Figures 8A–C).

## 5 Discussion

There is growing evidence that ROS is involved in all stages of tumorigenesis, including initiation, promotion, and progression (Weitzman and Gordon, 1990). Chronic oxidative stress has been proved to increase the invasiveness of the breast cancer cell line MCF-7 *in vitro* (Mahalingaiah et al., 2015), and mitochondrial DNA damage derived from OS could promote progression and metastasis in BC mouse models (Yuzefovych et al., 2016). All these suggest that oxidative stress occurs during the progression and metastasis of BC. As in TNBC, Malik et al. (2021) observed increased ROS levels in all the TNBC cell lines compared to normal cells and ER + breast cancer cell lines. In addition, the activated C/EBPβ/AEP signaling involved in OS-mediated metastasis in the TNBC cell line has been revealed by Lei et al. (2021) recently. However, more existing studies mainly focus on the induction of ROS by peroxidative drugs to cause the death of BC cells (Morotti et al., 2021), while the mechanism of ROS in the development of TNBC patients remains unclear. Despite the high recurrence rate and poor prognosis of TNBC patients, some sets of this population have the same prognosis as other subtypes (Liedtke et al., 2008), which should remind us that TNBC is a highly heterogeneous tumor, and gene expression profiling of individuals should be taken into consideration before risk-stratified treatment.

Fortunately, the development of sequencing technologies provides more platforms and opportunities to dissect the disease at the molecular level (Karaayvaz et al., 2018). As shown in our study, two different molecular subtypes were identified by consistent cluster analysis in TNBC datasets based on the prognosis-related OS genes, demonstrating differential prognosis and TME profiling, which also highlight the heterogeneity of TNBC patients. Although ROS can mediate the reprogramming of the extracellular matrix, cancer-associated fibroblasts, and endothelial cells in the breast TME (Wishart et al., 2020; Zheng et al., 2022), we did not observe any differences between the two molecular subtypes. Different TNBC datasets and different cell composition ratios in the breast TME may have explained this result, which also does not imply an opposite function driven by ROS in stromal cells. Cluster A is more enriched with tumor-infiltrating lymphocytes (TILs) than cluster B, which might contribute to a better RFS. Interestingly, some tumor-suppressor immune cells (Li et al., 2018), such as Tregs, are also enriched in cluster A. Operation plus systematic chemotherapy has been considered the standard regimen for non-metastatic TNBC (Brewster et al., 2014); however, the unsatisfactory treatment response has led researchers to focus on immunotherapy, a promising therapy that has achieved success in hematological diseases and several solid tumors (Brown and Bicknell, 2001; Nourazarian et al., 2014; Deepak et al., 2020). Previous studies showed that TNBC has more TILs and higher expression of PD-L1 in both immune and cancer cells than other tumors (Wilkerson and Hayes, 2010; Wu et al., 2021), which predict better immunotherapy response, making this subtype likely to benefit from immunotherapy. Unfortunately, we did not obtain a positive result from comparing single-agent pembrolizumab versus single-agent chemotherapy in the phase III KEYNOTE-119 trial (Weitzman and Gordon, 1990). Therefore, it is important to explore new molecular markers to select the potential population. Recently, some scholars have shown the expression of HLA-A and HLA-B was correlated with the markers of T-cell activation and the favorable prognosis in basal-like BC patients (Noblejas-López et al., 2019); these could be new markers for identifying better immunotherapy responses. Another study showed that HLA-I loss of heterozygosity (LOH) (loss of at least one of the HLA-A, HLA-B, and HLA-C genes) was an independent risk factor for RFS and worse immunotherapy response in TNBC patients (Zhou et al., 2021), which was consistent with our result. In addition, higher expression of important immune checkpoints, like PDCD1, PDL1, and CTLA4 (Yin et al., 2020), can be observed in molecular subtype A. All these observations suggest that our molecular subtypes based on OS-related genes could be applied to predict the efficacy of immunotherapy for TNBC patients.

To construct an OS-related prognostic signature for TNBC patients, eight hub OS genes (*PDCD1*, *CSF2*, *IL-6*, *AGTR1*, *SERPINA1*, *CYP27A1*, *GCLC*, and *KNG1*) were selected among the DEGs between clusters A and B via machine learning algorithms and the Cox regression model. The eight-gene signature was highly accurate in predicting the (1-, 3-, and 5-year) RFS for TNBC patients due to the high AUC values of tROC curves in the training and testing datasets. These genes have been shown to be associated with oxidative stress and involved in cancer progression. Programmed cell death 1 (PD1, also termed PDCD1) is an immunosuppressive molecule expressed in activated T cells as a marker of exhaustion and has been used to predict immunotherapy response. Wang et al. (2019) found that PD1 was upregulated and served as a risk factor in the immunomodulatory subtype of TNBC. On the other hand, the expression of PD1 on activated T cells can also limit oxidative metabolism (Bettonville et al., 2018), which was related to decreased generation of ROS, to promote T-cell survival and functional fitness. *CSF2*, known as GM-CSF, can stimulate GM and dendritic cell differentiation, and enhance the antigen-presenting function for antitumors (Yan et al., 2017). IL-6 takes part in various immune and inflammation procedures, and the inhibition of IL-6 expression can decrease colony formation and tumor growth in TNBC through NF-κB signaling (Hartman et al., 2013). However, the exact regulatory mechanism of ROS/IL-6 in TNBC remains unclear. As for another risk gene, *AGTR1*, previous studies already revealed that *AGTR1* can promote the metastasis and invasion of breast cancer via the CXCR4/SDF-1α axis (Singh et al., 2020), and the AGTR1 KO TNBC cell line demonstrated an attenuated EMT process (Moschetta-Pinheiro et al., 2021). On the contrary, *SERPINA1* (Chan et al., 2015) and *CYP27A1* (Kimbung et al., 2017) were considered favorable genes for prognosis in BC patients, and *GCLC* is involved in glutathione biosynthesis and could eliminate the irradiation-derived ROS via increasing the glutathione, thus driving resistance in TNBC (Bai et al., 2021). As for *KNG1*, an important pro-inflammatory and pro-oxidant factor, there are few studies on its role in breast cancer. Furthermore, based on the risk score of the signature, we included some significant risk variables, like age, T stage, and N stage, in the nomogram for comprehensively assessing the 1-, 3-, and 5-year RFS of TNBC patients, and it demonstrated effective predictive power, which may provide an easy and accurate method for clinicians to evaluate the RFS of TNBC patients.

The function analysis of DEGs between clusters A and B revealed that these genes are involved in some OS-related and immune activity biological processes, like the response to OS, T-cell activation, and positive regulation of cytokine production, as well as being predominantly enriched in the MAPK and TNF signaling pathways. For example, the MAPK signaling pathway is involved in directing cellular responses to stimuli, such as heat shock, ionophores, and pro-inflammatory cytokines (Seger and Krebs, 1995). Previous studies have shown that the activation of the MAPK/JUN pathway could induce ROS-mediated cell death in TNBC cell lines (Zhang et al., 2015; Dai et al., 2021); on the other hand, ROS derived from the pathway can also promote the aggressiveness of TNBC cells (Zhao et al., 2017). Meanwhile, to explore the interaction relationships and hub gene modules of these DEGs, we successfully identified three hub modules in the PPI network. These three modules included nine genes (*MT-ND3*, *MT-ND1*, *MT-ND4*, *RAC2*, *CYBA*, *NCF4*, *GRB2*, *SHC1*, and *PIK3R1*) shown to be involved in the progression and development of cancers or diseases (Jiang et al., 2018; Mani et al., 2019; Liu J. et al., 2022).

However, our study still lacks in some areas. First, the TME is a complicated context for the progression of cancers. Thus, the ROS-mediated crosstalk among immune cells, tumor cells, and stromal cells should be further explored to elaborate on the role of OS in the TNBC TME. Second, more immunotherapy datasets and multicenter randomized controlled trials are needed to validate our molecular subtypes and risk model in the TNBC population. Third, experiments should be further performed to explore the function of the hub OS genes.

## 6 Conclusion

In summary, our stratified OS-related molecular subtypes depict two different molecular clusters and immune profiles of TNBC, which is of great significance for predicting the prognosis and immunotherapy efficacy for TNBC patients. In addition, the OS-related signature and nomogram can provide important supplement risk evaluation to direct individual precision therapy.

## Data Availability

The datasets presented in this study can be found in online repositories. The names of the repository/repositories and accession number(s) can be found in the article/Supplementary Material.
