# Supplementary material for: Oxidative stress genes define two subtypes of triple-negative breast cancer with prognostic and therapeutic implications
Source: Front Genet. 2023 Jul 13;14:1230911. doi: 10.3389/fgene.2023.1230911 (PMC10372428; doi:10.3389/fgene.2023.1230911)
Supplement: Supplementary file 1 [file Table4.DOC]

**Supplement Table 4 | Eight OS-related prognostic genes significantly associated with RFS of TNBC patients.**

| **Gene** | **Coef** | **HR** | **HR.95L** | **HR.95H** | **P value** |
| --- | --- | --- | --- | --- | --- |
| PDCD1 | -0.329525354 | 0.719265048 | 0.491408383 | 1.05277449 | 0.090005672 |
| CSF2 | -0.316236293 | 0.728887199 | 0.466428323 | 1.139031493 | 0.16500868 |
| IL6 | 0.16509038 | 1.179499717 | 0.974012064 | 1.428339169 | 0.090959943 |
| AGTR1 | 0.232422544 | 1.26165272 | 0.977770072 | 1.62795695 | 0.073919957 |
| SERPINA1 | -0.220580168 | 0.802053337 | 0.633206925 | 1.015923121 | 0.067403345 |
| CYP27A1 | -0.285188297 | 0.751872666 | 0.541831515 | 1.043336332 | 0.087978014 |
| GCLC | -0.697833762 | 0.497662192 | 0.27496885 | 0.900711689 | 0.021142398 |
| KNG1 | -0.348399969 | 0.705816515 | 0.450679698 | 1.105390269 | 0.127961841 |
