# Supplementary figures and images for: Oxidative stress genes define two subtypes of triple-negative breast cancer with prognostic and therapeutic implications
Source: Front Genet. 2023 Jul 13;14:1230911. doi: 10.3389/fgene.2023.1230911 (PMC10372428; doi:10.3389/fgene.2023.1230911)

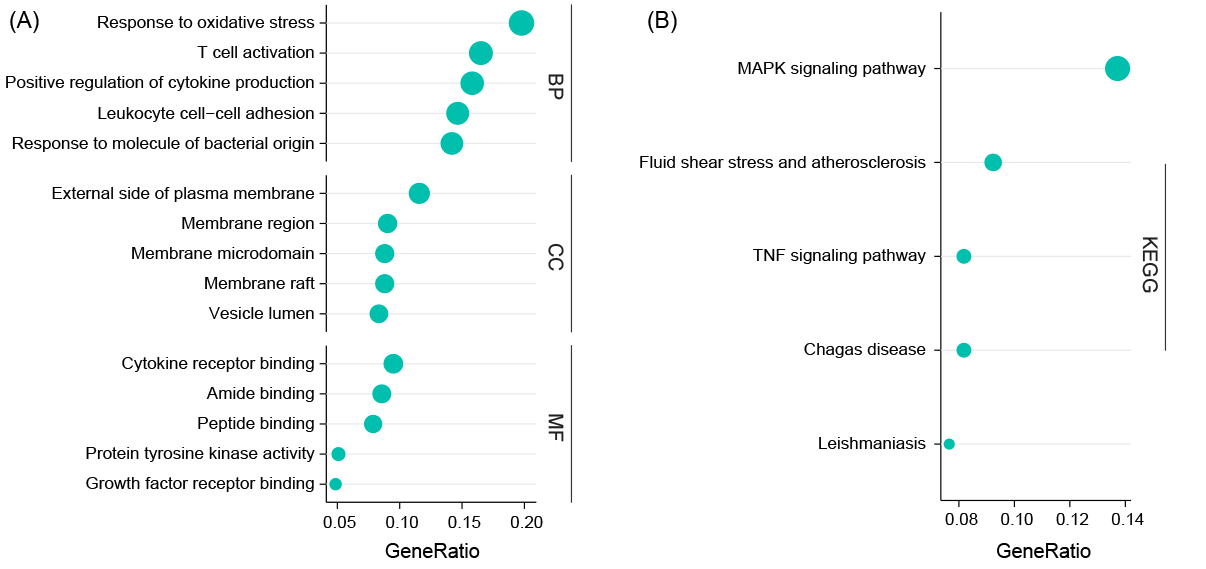

Supplement: Supplementary file 2 [file Image3.tif]

# consensus matrix k=2

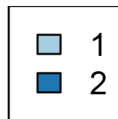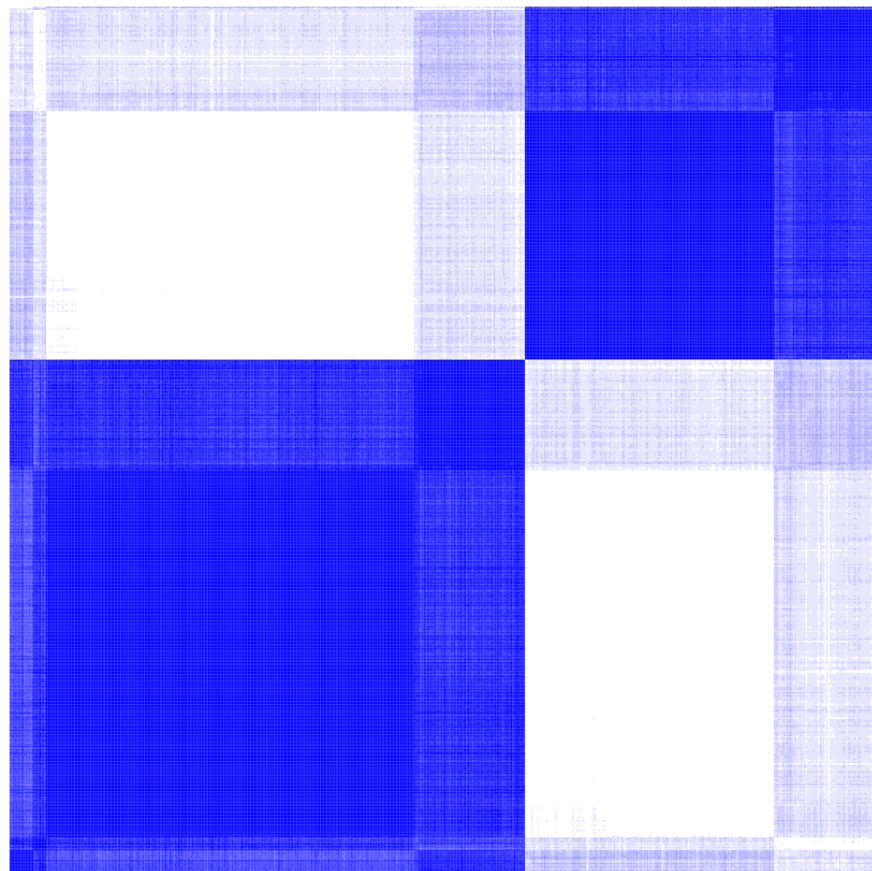

Supplement: Supplementary file 4 [file Image2.pdf]

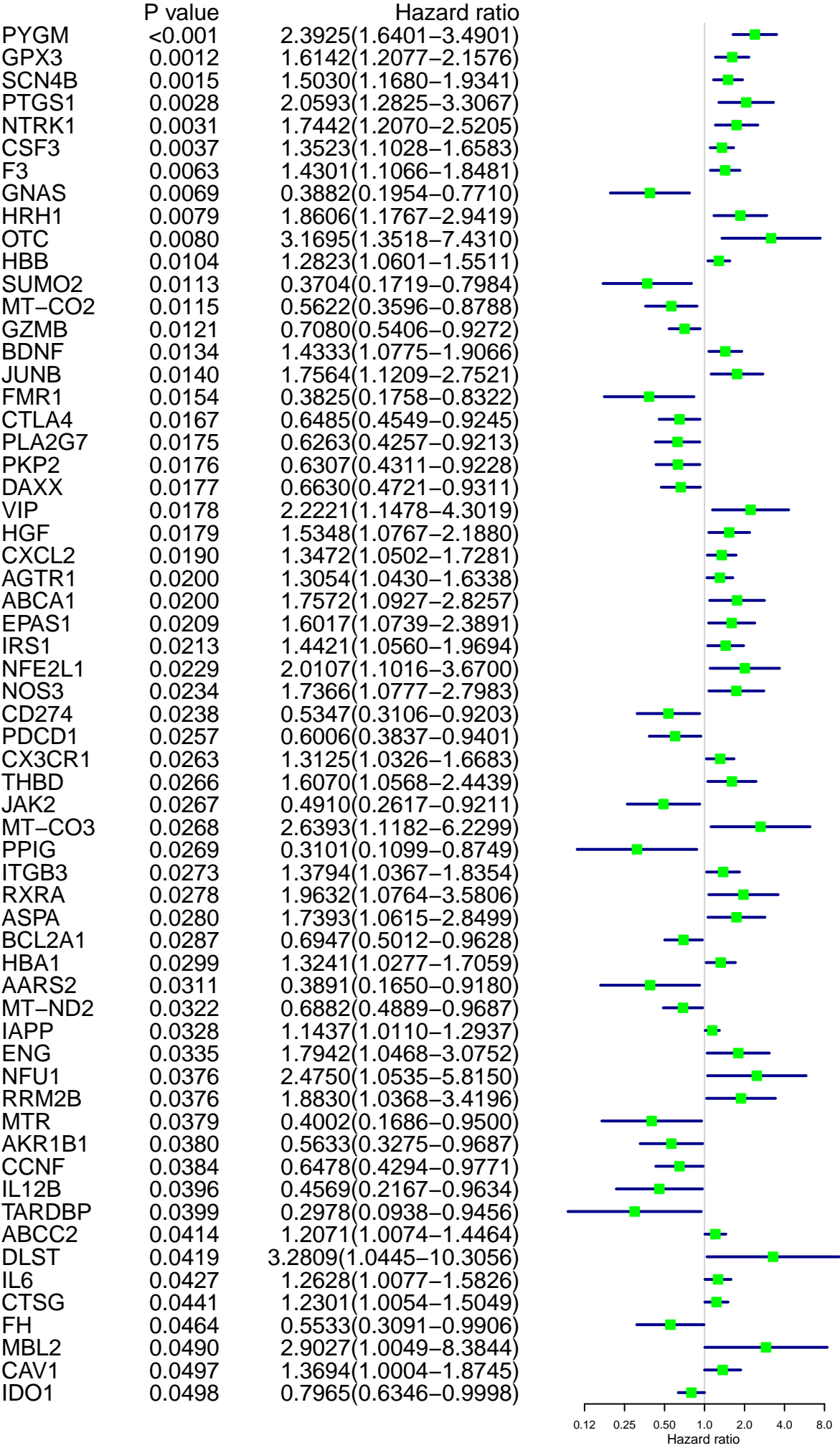

Supplement: Supplementary file 6 [file Image1.pdf]
