# Supplementary material for: Oxidative stress genes define two subtypes of triple-negative breast cancer with prognostic and therapeutic implications
Source: Front Genet. 2023 Jul 13;14:1230911. doi: 10.3389/fgene.2023.1230911 (PMC10372428; doi:10.3389/fgene.2023.1230911)
Supplement: Supplementary file 3 [file Table3.DOC]

**Supplement Table 3 | Univariate cox regression analysis showing associations between 27 OS-related genes and RFS in TNBC ( *p* <0.05).**

| Gene | HR | HR.95L | HR.95H | P value |
| --- | --- | --- | --- | --- |
| GZMB | 0.860442011 | 0.746611783 | 0.991627069 | 0.037884749 |
| CD274 | 0.743449235 | 0.575106107 | 0.961069199 | 0.023629763 |
| CTLA4 | 0.829230313 | 0.695122404 | 0.989211264 | 0.037481425 |
| PDCD1 | 0.630077238 | 0.476726757 | 0.832756542 | 0.001169995 |
| SOCS1 | 0.772023365 | 0.628857289 | 0.947782726 | 0.013419669 |
| CD40 | 0.716393276 | 0.537454808 | 0.954906941 | 0.02292703 |
| PLA2G7 | 0.720152598 | 0.578014688 | 0.897243227 | 0.003427533 |
| CSF2 | 0.691138683 | 0.487619115 | 0.979602039 | 0.03791554 |
| PDE5A | 1.447587719 | 1.081189957 | 1.938151748 | 0.012983274 |
| CD79A | 0.858582018 | 0.763805721 | 0.965118567 | 0.010622284 |
| IL6 | 1.177940477 | 1.004416408 | 1.381442753 | 0.043992594 |
| FOXP3 | 0.772542875 | 0.600101134 | 0.994536521 | 0.045233774 |
| PTPN1 | 0.594833137 | 0.357496458 | 0.989734174 | 0.04553419 |
| CAMK4 | 0.755350452 | 0.576150832 | 0.990286351 | 0.0422947 |
| NOX4 | 1.334622327 | 1.064100218 | 1.673918231 | 0.012505731 |
| GRB2 | 0.55957933 | 0.328962089 | 0.951869643 | 0.032197393 |
| AGTR1 | 1.206280998 | 1.017051699 | 1.43071768 | 0.031226474 |
| HRH1 | 1.397686461 | 1.038448633 | 1.881197954 | 0.027184216 |
| DLST | 2.512517075 | 1.289039266 | 4.897245737 | 0.006818084 |
| SERPINA1 | 0.775024335 | 0.646950717 | 0.928452051 | 0.00568341 |
| CD55 | 1.515968541 | 1.137475061 | 2.020405278 | 0.004527128 |
| AKR1B1 | 0.669374125 | 0.489445428 | 0.915447757 | 0.011970064 |
| CYP27A1 | 0.73064671 | 0.577138685 | 0.924984981 | 0.009107747 |
| INSR | 1.328844124 | 1.006873835 | 1.75377157 | 0.04460564 |
| CALR | 0.598847457 | 0.374261222 | 0.958203134 | 0.032517561 |
| GCLC | 0.622685922 | 0.415782691 | 0.932549061 | 0.021513169 |
| KNG1 | 0.687879918 | 0.493771972 | 0.958294127 | 0.02698014 |
