# Supplementary material for: Oxidative stress genes define two subtypes of triple-negative breast cancer with prognostic and therapeutic implications
Source: Front Genet. 2023 Jul 13;14:1230911. doi: 10.3389/fgene.2023.1230911 (PMC10372428; doi:10.3389/fgene.2023.1230911)
Supplement: Supplementary file 5 [file Table1.DOC]

**Supplement Table 1 | Clinical information of TNBC patients in this study.**

| **Variables** | **TCGA -BRCA** | **SRA-SRR8518252** |
| --- | --- | --- |
| **Total** | 188 | 360 |
| **Age** |  |  |
| <60 | 123 | 263 |
| ≥60 | 65 | 97 |
| **T stage** |  |  |
| T1 | 44 | 131  - |
| T2 | 120 | 219 |
| T3 | 16 | 9 |
| T4 | 7 | 1 |
| Unknown | 1 | -  - |
| **N stage** |  |  |
| N0 | 115 | 216 |
| N1 | 46 | 94 |
| N2 | 15 | 31 |
| N3 | 10 | 17 |
| Unknown | 2 | 2 |
| **RFS event** |  |  |
| Alive | 164 | 311 |
| Dead | 24 | 49 |

**Abbreviations:** TNBC, triple negative breast cancer ; TCGA, the cancer genome atlas; SRA, sequence read archive; T, tumor depth; N, lymph node metastasis; RFS,relapse-free survival.
