# Supplementary material for: Oxidative stress genes define two subtypes of triple-negative breast cancer with prognostic and therapeutic implications
Source: Front Genet. 2023 Jul 13;14:1230911. doi: 10.3389/fgene.2023.1230911 (PMC10372428; doi:10.3389/fgene.2023.1230911)
Supplement: Supplementary file 7 [file Table2.DOC]

**Supplement Table 2 | 441 oxidative stress-related differentially expressed genes between cluster A and cluster B in triple-negative breast cancer.**

| **Symbol** | **logFC** | **AveExpr** | **t** | **P.Value** | **adj.P.Val** |
| --- | --- | --- | --- | --- | --- |
| GZMB | -2.034477063 | 4.669195508 | -20.38397266 | 3.26E-69 | 4.14E-66 |
| IDO1 | -2.446252411 | 5.695935239 | -19.96318667 | 4.33E-67 | 2.75E-64 |
| BCL2A1 | -1.776511503 | 5.086038753 | -17.44292322 | 1.49E-54 | 5.97E-52 |
| CXCL10 | -2.225066119 | 7.979718915 | -17.42212209 | 1.88E-54 | 5.97E-52 |
| CD274 | -1.062421017 | 5.124047952 | -16.5877714 | 2.11E-50 | 5.37E-48 |
| IRF1 | -1.117871325 | 7.583532532 | -16.49891093 | 5.66E-50 | 1.20E-47 |
| STAT1 | -1.187004399 | 10.33012156 | -15.14191089 | 1.52E-43 | 2.54E-41 |
| IFNG | -1.564225521 | 2.737810324 | -15.13449705 | 1.65E-43 | 2.54E-41 |
| CTLA4 | -1.315201032 | 4.703791124 | -15.12653995 | 1.80E-43 | 2.54E-41 |
| FASLG | -1.31333076 | 3.200195332 | -14.87484101 | 2.65E-42 | 3.36E-40 |
| CD86 | -0.841053017 | 6.191248039 | -14.79377082 | 6.28E-42 | 7.25E-40 |
| TLR8 | -0.994633061 | 4.740558513 | -14.45542342 | 2.25E-40 | 2.38E-38 |
| CD38 | -1.468428824 | 5.597955929 | -14.37655693 | 5.15E-40 | 5.03E-38 |
| CD80 | -0.725036598 | 4.526593152 | -13.83577687 | 1.43E-37 | 1.30E-35 |
| CCR5 | -1.064476437 | 5.84213233 | -13.7552773 | 3.28E-37 | 2.78E-35 |
| CASP1 | -0.776107068 | 6.410229399 | -13.59770663 | 1.65E-36 | 1.31E-34 |
| TYMP | -0.987464368 | 8.657729405 | -13.4887031 | 5.03E-36 | 3.76E-34 |
| NCF1 | -0.815644812 | 4.385187088 | -13.38193997 | 1.49E-35 | 1.05E-33 |
| IL2RA | -1.036154899 | 5.017883572 | -13.15803161 | 1.43E-34 | 9.58E-33 |
| CXCL9 | -1.894981849 | 7.915797319 | -12.97103444 | 9.34E-34 | 5.93E-32 |
| ICAM1 | -1.029139616 | 7.59618194 | -12.92032299 | 1.55E-33 | 9.37E-32 |
| VIPR1 | 1.241239284 | 4.70559939 | 12.90387578 | 1.83E-33 | 1.05E-31 |
| IL15 | -0.722367162 | 5.102384355 | -12.89546353 | 1.98E-33 | 1.10E-31 |
| SOD2 | -0.931703508 | 9.828707554 | -12.86773459 | 2.62E-33 | 1.38E-31 |
| SCN4B | 1.037952946 | 3.73280912 | 12.46538614 | 1.38E-31 | 7.03E-30 |
| PDCD1 | -1.009755026 | 1.050026392 | -12.40978712 | 2.38E-31 | 1.16E-29 |
| SOCS1 | -1.002259584 | 4.213540494 | -12.17392055 | 2.35E-30 | 1.11E-28 |
| CXCR3 | -1.140995712 | 4.03637528 | -12.07851372 | 5.89E-30 | 2.67E-28 |
| CD40 | -0.73785103 | 5.91164435 | -12.02932552 | 9.45E-30 | 4.14E-28 |
| LCK | -1.002383152 | 5.589872153 | -12.0033085 | 1.21E-29 | 5.13E-28 |
| CYBB | -0.9726099 | 8.571864652 | -11.96717359 | 1.71E-29 | 7.02E-28 |
| CYBA | -0.832506965 | 8.189645111 | -11.9310912 | 2.42E-29 | 9.60E-28 |
| ISG15 | -1.334601614 | 8.040793274 | -11.54509236 | 9.29E-28 | 3.58E-26 |
| CD69 | -0.982616357 | 5.037267449 | -11.5204556 | 1.17E-27 | 4.37E-26 |
| TNFAIP3 | -0.709136902 | 7.736765801 | -11.50582977 | 1.34E-27 | 4.87E-26 |
| IL2RB | -1.038840471 | 5.533920966 | -11.34373915 | 6.06E-27 | 2.14E-25 |
| NCF4 | -0.740500153 | 4.655547849 | -11.33319736 | 6.68E-27 | 2.29E-25 |
| EDNRA | 0.822112449 | 6.29439017 | 11.23245783 | 1.69E-26 | 5.66E-25 |
| B2M | -1.034467351 | 10.87833127 | -11.19776752 | 2.33E-26 | 7.58E-25 |
| IL18 | -0.648553914 | 5.915296451 | -10.9035219 | 3.40E-25 | 1.08E-23 |
| CLEC4A | -0.628560984 | 4.358854331 | -10.8541749 | 5.31E-25 | 1.65E-23 |
| PRKG1 | 0.690186418 | 5.606122544 | 10.61020912 | 4.71E-24 | 1.43E-22 |
| PTPRC | -0.909935657 | 7.908640961 | -10.59853898 | 5.23E-24 | 1.54E-22 |
| NCF2 | -0.670554716 | 6.628545512 | -10.58356955 | 5.97E-24 | 1.72E-22 |
| OPTN | -0.64254649 | 8.353951981 | -10.55250498 | 7.86E-24 | 2.22E-22 |
| TNFRSF1B | -0.710510138 | 7.172324285 | -10.54849496 | 8.15E-24 | 2.25E-22 |
| PLA2G7 | -0.850239661 | 5.996464198 | -10.54333534 | 8.53E-24 | 2.30E-22 |
| MSRB3 | 0.803685367 | 6.561271943 | 10.40050965 | 3.00E-23 | 7.94E-22 |
| PTPN22 | -0.626962746 | 5.645262725 | -10.36933878 | 3.94E-23 | 1.02E-21 |
| TGFB3 | 0.808575533 | 6.761219468 | 10.33896922 | 5.14E-23 | 1.31E-21 |
| ITGAM | -0.611727657 | 6.554183964 | -10.29834196 | 7.33E-23 | 1.83E-21 |
| VCAM1 | -0.895258147 | 7.271175166 | -10.29349232 | 7.65E-23 | 1.87E-21 |
| TLR6 | -0.617075245 | 5.911517187 | -10.18778063 | 1.91E-22 | 4.59E-21 |
| STAT4 | -0.679280272 | 5.215295471 | -10.18344986 | 1.99E-22 | 4.67E-21 |
| FCGR3A | -0.789670352 | 8.305746787 | -10.00024953 | 9.60E-22 | 2.22E-20 |
| AR | 1.777644852 | 6.301216924 | 9.782696209 | 6.07E-21 | 1.38E-19 |
| PTK2B | -0.623631152 | 7.137155125 | -9.766695839 | 6.95E-21 | 1.55E-19 |
| HPX | 1.067291222 | 3.806883288 | 9.756162784 | 7.59E-21 | 1.66E-19 |
| SUOX | 0.47738771 | 6.012652451 | 9.751932076 | 7.87E-21 | 1.69E-19 |
| CIITA | -0.741188738 | 7.45875233 | -9.726015746 | 9.78E-21 | 2.07E-19 |
| GCH1 | -0.598576108 | 6.150196911 | -9.636698829 | 2.06E-20 | 4.29E-19 |
| CACNA1C | 0.639953065 | 6.004730099 | 9.545430519 | 4.40E-20 | 9.02E-19 |
| IGF2 | 1.388916044 | 5.930146068 | 9.528168627 | 5.08E-20 | 1.02E-18 |
| GSTM3 | 1.100397793 | 6.027572255 | 9.505373105 | 6.13E-20 | 1.22E-18 |
| GSK3B | 0.331336596 | 8.139668502 | 9.453880413 | 9.37E-20 | 1.83E-18 |
| ALAD | 0.371917033 | 6.817317858 | 9.402992639 | 1.42E-19 | 2.74E-18 |
| JAK2 | -0.505559552 | 7.247461356 | -9.388483925 | 1.60E-19 | 3.03E-18 |
| PRKD1 | 0.568893125 | 5.069587037 | 9.192020224 | 7.89E-19 | 1.47E-17 |
| CD34 | 0.590953908 | 6.35519355 | 9.186456319 | 8.26E-19 | 1.52E-17 |
| HLA-DRA | -1.090667936 | 4.777790528 | -9.157467916 | 1.04E-18 | 1.89E-17 |
| SCO2 | -0.566884441 | 5.825874788 | -9.136174741 | 1.24E-18 | 2.21E-17 |
| BAK1 | -0.425097696 | 6.480638957 | -9.090760188 | 1.78E-18 | 3.14E-17 |
| LYN | -0.550772088 | 7.256676039 | -9.051520887 | 2.43E-18 | 4.21E-17 |
| ITGAL | -0.81482751 | 6.299828216 | -9.050602937 | 2.45E-18 | 4.21E-17 |
| BACE1 | 0.382249784 | 6.819528823 | 9.032128506 | 2.84E-18 | 4.81E-17 |
| RAC2 | -0.764534039 | 6.65534272 | -8.999630101 | 3.68E-18 | 6.15E-17 |
| BAG3 | 0.492775445 | 7.074034728 | 8.994036658 | 3.84E-18 | 6.28E-17 |
| SORCS2 | 0.781986219 | 4.146588671 | 8.993600262 | 3.86E-18 | 6.28E-17 |
| NEIL1 | 0.529248852 | 5.598376822 | 8.982285763 | 4.22E-18 | 6.78E-17 |
| ITGB2 | -0.669419659 | 8.160539434 | -8.970766657 | 4.62E-18 | 7.34E-17 |
| RARA | 0.585137101 | 6.474383625 | 8.958313049 | 5.10E-18 | 8.00E-17 |
| IL10 | -0.616523977 | 2.942921319 | -8.946260943 | 5.61E-18 | 8.69E-17 |
| PDGFRB | 0.688701769 | 8.629445277 | 8.891934523 | 8.62E-18 | 1.32E-16 |
| CEBPB | -0.603950521 | 8.139230821 | -8.795579765 | 1.83E-17 | 2.77E-16 |
| DNASE1 | 0.388876856 | 6.347837485 | 8.793245381 | 1.87E-17 | 2.79E-16 |
| TPPP3 | 0.714157261 | 4.578121415 | 8.775993659 | 2.14E-17 | 3.15E-16 |
| TRIM21 | -0.455606829 | 6.474990671 | -8.748627731 | 2.64E-17 | 3.86E-16 |
| TPO | 0.987440151 | 2.76787137 | 8.73905376 | 2.85E-17 | 4.11E-16 |
| CSF2 | -0.587355843 | 0.61098344 | -8.734892063 | 2.94E-17 | 4.20E-16 |
| PRKAA1 | 0.401943892 | 7.962915123 | 8.732401655 | 3.00E-17 | 4.23E-16 |
| FCGR2B | -0.547726125 | 5.538462542 | -8.730494726 | 3.04E-17 | 4.25E-16 |
| PDE5A | 0.483473712 | 6.657747422 | 8.629920604 | 6.62E-17 | 9.14E-16 |
| SELL | -1.015784459 | 6.023475092 | -8.624415575 | 6.91E-17 | 9.44E-16 |
| TSC2 | 0.356080206 | 7.676610016 | 8.606876575 | 7.91E-17 | 1.07E-15 |
| EPHX1 | 0.594151906 | 8.084654487 | 8.588757468 | 9.09E-17 | 1.22E-15 |
| GSTO1 | -0.359246491 | 7.6648038 | -8.499817573 | 1.79E-16 | 2.37E-15 |
| CCR7 | -0.961894494 | 4.96560072 | -8.496596054 | 1.84E-16 | 2.41E-15 |
| SOCS3 | -0.655704706 | 7.642874645 | -8.469246778 | 2.26E-16 | 2.93E-15 |
| TSC1 | 0.300330319 | 7.230482558 | 8.449757751 | 2.62E-16 | 3.37E-15 |
| PRKCB | -0.782433359 | 5.66954443 | -8.442512082 | 2.77E-16 | 3.52E-15 |
| CYB5R3 | 0.39226255 | 8.15056566 | 8.410815549 | 3.52E-16 | 4.43E-15 |
| NOSTRIN | 0.750076614 | 4.405405631 | 8.386834099 | 4.22E-16 | 5.26E-15 |
| MT-CYB | 0.603366807 | 11.96422603 | 8.347084338 | 5.69E-16 | 7.02E-15 |
| ACOX2 | 0.820376101 | 4.0636999 | 8.341329574 | 5.94E-16 | 7.26E-15 |
| NOD2 | -0.544792752 | 5.348606885 | -8.328849491 | 6.53E-16 | 7.90E-15 |
| CD4 | -0.589614142 | 7.482271579 | -8.324655743 | 6.74E-16 | 8.07E-15 |
| MAPK10 | 1.0435187 | 4.922982753 | 8.317889544 | 7.09E-16 | 8.41E-15 |
| IL12B | -0.693410513 | 1.928564085 | -8.313349211 | 7.33E-16 | 8.62E-15 |
| HPSE | -0.505353602 | 5.005358054 | -8.309451533 | 7.55E-16 | 8.80E-15 |
| MMP7 | -1.651132913 | 8.036443045 | -8.284830954 | 9.08E-16 | 1.05E-14 |
| FGF1 | 0.651023742 | 5.120760469 | 8.274450711 | 9.81E-16 | 1.12E-14 |
| ALOX5 | -0.864455945 | 3.787441136 | -8.244841175 | 1.22E-15 | 1.39E-14 |
| EGF | 1.284928592 | 4.429368904 | 8.193091401 | 1.80E-15 | 2.02E-14 |
| FAS | -0.513414652 | 6.517473346 | -8.135530877 | 2.75E-15 | 3.06E-14 |
| IRF5 | -0.412218893 | 5.733146476 | -8.130851736 | 2.84E-15 | 3.14E-14 |
| PFN1 | -0.32135354 | 10.01372727 | -8.114982361 | 3.20E-15 | 3.50E-14 |
| ERBB4 | 1.366285419 | 4.06928779 | 8.10627819 | 3.41E-15 | 3.70E-14 |
| MAPT | 0.922185681 | 1.385356014 | 8.048835223 | 5.19E-15 | 5.59E-14 |
| ABCC8 | 1.195233449 | 1.675512504 | 8.012329249 | 6.77E-15 | 7.23E-14 |
| IL23A | -0.595212039 | 3.092712785 | -8.001816041 | 7.31E-15 | 7.74E-14 |
| CD79A | -1.26704649 | 5.292444318 | -7.980890145 | 8.51E-15 | 8.94E-14 |
| KLRK1 | -0.611210315 | 2.891323747 | -7.909379671 | 1.43E-14 | 1.49E-13 |
| PLCB1 | 0.625895978 | 6.20238902 | 7.862496324 | 2.00E-14 | 2.07E-13 |
| SLC22A5 | 0.40721043 | 6.051445771 | 7.835675382 | 2.43E-14 | 2.48E-13 |
| NFKBIA | -0.397738982 | 8.890994937 | -7.818907624 | 2.73E-14 | 2.78E-13 |
| IRAK1 | -0.492350385 | 8.88427859 | -7.798742032 | 3.16E-14 | 3.18E-13 |
| PXN | 0.444164262 | 7.380504144 | 7.770774012 | 3.85E-14 | 3.85E-13 |
| BIRC5 | -0.785676016 | 7.084652648 | -7.764248363 | 4.04E-14 | 4.01E-13 |
| ABL1 | 0.307539408 | 7.969025766 | 7.740355756 | 4.78E-14 | 4.71E-13 |
| ABCG2 | 0.536461741 | 3.640878791 | 7.734460153 | 4.99E-14 | 4.87E-13 |
| CX3CR1 | 0.687537792 | 4.025044925 | 7.707049978 | 6.06E-14 | 5.87E-13 |
| MT-ND5 | 0.630455648 | 11.53918737 | 7.68076764 | 7.29E-14 | 7.02E-13 |
| KDR | 0.437507496 | 6.984705747 | 7.654881043 | 8.75E-14 | 8.35E-13 |
| ATXN2 | 0.259026993 | 7.28841748 | 7.625244654 | 1.08E-13 | 1.02E-12 |
| CREBBP | 0.329793096 | 8.311055607 | 7.610542979 | 1.19E-13 | 1.12E-12 |
| SLC8A1 | -0.386375878 | 6.25103224 | -7.555124805 | 1.76E-13 | 1.64E-12 |
| CCL2 | -0.702485321 | 6.465696418 | -7.549297536 | 1.83E-13 | 1.70E-12 |
| MT-ATP6 | 0.554498788 | 11.87419393 | 7.535203807 | 2.02E-13 | 1.86E-12 |
| CDH5 | 0.482745578 | 6.557093126 | 7.46807254 | 3.21E-13 | 2.94E-12 |
| TPI1 | -0.442703013 | 10.36226278 | -7.425371678 | 4.31E-13 | 3.91E-12 |
| FMO4 | 0.637994369 | 5.275507464 | 7.422060361 | 4.41E-13 | 3.97E-12 |
| IL1RAPL2 | 0.974187679 | 1.688805831 | 7.420387373 | 4.46E-13 | 3.99E-12 |
| HSPG2 | 0.578169842 | 9.315505999 | 7.419541028 | 4.49E-13 | 3.99E-12 |
| BTD | 0.337448451 | 5.744839518 | 7.375444973 | 6.07E-13 | 5.35E-12 |
| AURKA | -0.616621813 | 6.487271367 | -7.336951414 | 7.89E-13 | 6.91E-12 |
| IL6 | -0.814428154 | 2.873579174 | -7.331039761 | 8.21E-13 | 7.14E-12 |
| PRDM10 | 0.212917896 | 5.978226744 | 7.326152091 | 8.49E-13 | 7.34E-12 |
| ITGA2 | 0.60989711 | 6.926530754 | 7.307819722 | 9.62E-13 | 8.25E-12 |
| ETFDH | 0.299465247 | 6.133086563 | 7.273990686 | 1.21E-12 | 1.03E-11 |
| EPHA3 | 0.680037088 | 4.031375928 | 7.265737143 | 1.28E-12 | 1.08E-11 |
| BMP4 | 0.853101947 | 3.532021321 | 7.243188098 | 1.49E-12 | 1.25E-11 |
| SELE | -0.874729415 | 4.456097798 | -7.21262107 | 1.83E-12 | 1.53E-11 |
| CCNA2 | -0.598008664 | 6.799793817 | -7.194544893 | 2.06E-12 | 1.71E-11 |
| DCTN1 | 0.276588607 | 8.384596113 | 7.168814652 | 2.45E-12 | 2.02E-11 |
| PIK3CG | -0.469578305 | 5.846831342 | -7.154655547 | 2.69E-12 | 2.21E-11 |
| CSF1 | -0.50356076 | 6.842755329 | -7.125460571 | 3.27E-12 | 2.66E-11 |
| IGF2BP2 | -1.060324928 | 5.127714098 | -7.101561327 | 3.83E-12 | 3.10E-11 |
| MSN | -0.42722929 | 10.20027752 | -7.089227517 | 4.16E-12 | 3.33E-11 |
| AOC3 | 0.644022847 | 5.561318745 | 7.089070902 | 4.16E-12 | 3.33E-11 |
| PLA2G6 | 0.398378758 | 5.474207162 | 7.087478134 | 4.21E-12 | 3.34E-11 |
| BDKRB2 | 0.50622763 | 4.774269324 | 7.082668189 | 4.34E-12 | 3.43E-11 |
| RXRA | 0.390432406 | 7.184178013 | 7.022842606 | 6.45E-12 | 5.05E-11 |
| CYGB | 0.462127819 | 4.680198398 | 7.01953441 | 6.59E-12 | 5.13E-11 |
| GPX1 | -0.36168056 | 9.129183133 | -6.953525465 | 1.01E-11 | 7.86E-11 |
| PCNA | -0.41424144 | 8.64062048 | -6.8882533 | 1.55E-11 | 1.19E-10 |
| ERBB2 | 0.838705169 | 8.882452892 | 6.885629198 | 1.58E-11 | 1.21E-10 |
| CACNB4 | 0.561117896 | 4.650933861 | 6.880819665 | 1.63E-11 | 1.24E-10 |
| TRAF2 | -0.41261915 | 6.799331723 | -6.872189276 | 1.72E-11 | 1.30E-10 |
| GLRX | -0.406860291 | 6.458159427 | -6.863654729 | 1.82E-11 | 1.37E-10 |
| APOE | -0.652292866 | 10.09449171 | -6.856056843 | 1.91E-11 | 1.43E-10 |
| EIF2AK2 | -0.394477664 | 8.669375397 | -6.84646858 | 2.03E-11 | 1.51E-10 |
| IRS1 | 0.581235401 | 6.428358087 | 6.845778109 | 2.04E-11 | 1.51E-10 |
| FASN | 0.857210729 | 9.99268024 | 6.831306613 | 2.24E-11 | 1.64E-10 |
| REN | 0.805719944 | 1.512323036 | 6.814420867 | 2.49E-11 | 1.82E-10 |
| CYP20A1 | 0.222044595 | 6.178273078 | 6.806201058 | 2.63E-11 | 1.91E-10 |
| LBR | -0.383364347 | 7.836171046 | -6.792589037 | 2.87E-11 | 2.07E-10 |
| CXCL16 | -0.475406461 | 7.465590103 | -6.788686499 | 2.94E-11 | 2.11E-10 |
| HADH | 0.360111239 | 7.328687065 | 6.772894876 | 3.25E-11 | 2.32E-10 |
| PLAT | 0.622950057 | 6.71751763 | 6.765484816 | 3.41E-11 | 2.42E-10 |
| EZH2 | -0.484781572 | 7.36619319 | -6.758841663 | 3.56E-11 | 2.51E-10 |
| TLR2 | -0.459801206 | 6.897478509 | -6.753141853 | 3.69E-11 | 2.59E-10 |
| ATXN3 | 0.219794662 | 6.594610333 | 6.733353175 | 4.18E-11 | 2.91E-10 |
| CD40LG | -0.573345504 | 3.080888531 | -6.732765386 | 4.20E-11 | 2.91E-10 |
| NR3C2 | 0.472008926 | 5.354392891 | 6.729863167 | 4.28E-11 | 2.95E-10 |
| MYLK | 0.570211292 | 8.057852759 | 6.714267105 | 4.72E-11 | 3.24E-10 |
| CYP2A6 | 0.682362053 | 2.889959799 | 6.692866269 | 5.41E-11 | 3.69E-10 |
| OSM | -0.677578651 | 3.875437809 | -6.667975388 | 6.33E-11 | 4.30E-10 |
| IL1B | -0.531232939 | 4.331211231 | -6.649241777 | 7.12E-11 | 4.81E-10 |
| HSPB1 | 0.702042933 | 8.863485974 | 6.6474006 | 7.20E-11 | 4.84E-10 |
| FOXP3 | -0.483596411 | 4.33580098 | -6.633655714 | 7.85E-11 | 5.25E-10 |
| PINK1 | 0.288497369 | 5.594093334 | 6.630518946 | 8.01E-11 | 5.32E-10 |
| CRAT | 0.833972312 | 6.742452998 | 6.624076229 | 8.34E-11 | 5.51E-10 |
| CCNB1 | -0.503737125 | 7.375195016 | -6.617171997 | 8.71E-11 | 5.73E-10 |
| ACAD8 | 0.340176046 | 6.364405411 | 6.61377694 | 8.89E-11 | 5.82E-10 |
| HLA-B | -1.100587446 | 9.149563982 | -6.55840433 | 1.26E-10 | 8.18E-10 |
| FOXM1 | -0.690946857 | 6.99327936 | -6.552989505 | 1.30E-10 | 8.39E-10 |
| IGF1R | 0.617607581 | 7.783610123 | 6.552760727 | 1.30E-10 | 8.39E-10 |
| GAPDH | -0.476715681 | 12.14746298 | -6.549764109 | 1.33E-10 | 8.50E-10 |
| AKT1 | 0.363630884 | 8.235406847 | 6.530661239 | 1.49E-10 | 9.52E-10 |
| TXNRD2 | 0.293983213 | 5.931380405 | 6.528978371 | 1.51E-10 | 9.58E-10 |
| CDKN1B | -0.381584555 | 8.029441407 | -6.525935917 | 1.54E-10 | 9.71E-10 |
| PTPN1 | -0.243830428 | 7.932390152 | -6.507932488 | 1.72E-10 | 1.08E-09 |
| NLRP3 | -0.385209102 | 4.861133535 | -6.488630353 | 1.93E-10 | 1.21E-09 |
| MT-ND6 | 0.583836165 | 9.707478576 | 6.480577059 | 2.03E-10 | 1.27E-09 |
| MECOM | 0.444706868 | 5.566346672 | 6.468364154 | 2.19E-10 | 1.36E-09 |
| SMPD1 | 0.327817594 | 6.405921725 | 6.465123016 | 2.24E-10 | 1.38E-09 |
| RPS6KA5 | 0.376443792 | 6.044119907 | 6.455222221 | 2.38E-10 | 1.46E-09 |
| PML | -0.334905991 | 7.658375111 | -6.451744606 | 2.43E-10 | 1.48E-09 |
| ADH1C | 1.033496634 | 2.327322417 | 6.44576151 | 2.52E-10 | 1.53E-09 |
| CAMK4 | -0.467952218 | 5.043916568 | -6.443385948 | 2.56E-10 | 1.55E-09 |
| ANGPT2 | 0.314210708 | 5.983941113 | 6.43154482 | 2.75E-10 | 1.65E-09 |
| NDUFA9 | -0.348418406 | 6.294570911 | -6.4199781 | 2.95E-10 | 1.76E-09 |
| TIMP2 | 0.485032729 | 9.053252056 | 6.419818718 | 2.95E-10 | 1.76E-09 |
| CTSB | -0.37151406 | 11.07839553 | -6.418615862 | 2.97E-10 | 1.76E-09 |
| SLC40A1 | 0.722591768 | 7.998969037 | 6.413263633 | 3.07E-10 | 1.81E-09 |
| TAC1 | 0.530349093 | 0.619321237 | 6.40203756 | 3.29E-10 | 1.93E-09 |
| SPARC | 0.557557424 | 11.80270201 | 6.38563156 | 3.64E-10 | 2.13E-09 |
| GRIN1 | 0.79290268 | 3.192369923 | 6.378495117 | 3.80E-10 | 2.21E-09 |
| ETS1 | -0.434348044 | 8.698351066 | -6.376003538 | 3.85E-10 | 2.24E-09 |
| PDGFB | 0.455278592 | 6.460530509 | 6.358322116 | 4.29E-10 | 2.48E-09 |
| CXCR4 | -0.514548782 | 8.601519613 | -6.345276004 | 4.64E-10 | 2.67E-09 |
| CDKN2A | -0.953412996 | 6.138388074 | -6.337570227 | 4.87E-10 | 2.78E-09 |
| ABCC2 | 0.828560844 | 4.231169979 | 6.335099081 | 4.94E-10 | 2.81E-09 |
| APC | 0.233785236 | 7.284835187 | 6.320408457 | 5.40E-10 | 3.06E-09 |
| CYB5A | 0.569458886 | 6.882295406 | 6.299857759 | 6.11E-10 | 3.45E-09 |
| SYK | -0.389887818 | 7.34865154 | -6.292307361 | 6.39E-10 | 3.59E-09 |
| MT-CO3 | 0.3789743 | 13.79345837 | 6.291372264 | 6.43E-10 | 3.60E-09 |
| HLA-A | -1.16398197 | 7.346885579 | -6.275514824 | 7.07E-10 | 3.94E-09 |
| FAAH | 0.485711642 | 5.97546564 | 6.274574928 | 7.11E-10 | 3.94E-09 |
| SIRT3 | 0.260968362 | 5.673774961 | 6.254088458 | 8.04E-10 | 4.44E-09 |
| KIAA0319L | 0.262324814 | 7.893811406 | 6.24918341 | 8.28E-10 | 4.55E-09 |
| GPX8 | 0.429438822 | 6.815357475 | 6.220099277 | 9.85E-10 | 5.39E-09 |
| SCP2 | 0.410780695 | 8.114568869 | 6.212661226 | 1.03E-09 | 5.61E-09 |
| GAL | -1.018078776 | 3.350417413 | -6.206108051 | 1.07E-09 | 5.81E-09 |
| FLT1 | 0.325727752 | 7.073693507 | 6.18877574 | 1.19E-09 | 6.41E-09 |
| MLYCD | 0.249285599 | 5.887157899 | 6.184648999 | 1.22E-09 | 6.54E-09 |
| HMGCR | 0.324638305 | 7.31786381 | 6.138371965 | 1.60E-09 | 8.56E-09 |
| FCGR2A | -0.381342408 | 7.285332026 | -6.115397219 | 1.83E-09 | 9.76E-09 |
| EP300 | 0.262093335 | 8.679638129 | 6.103455602 | 1.96E-09 | 1.04E-08 |
| KCNMA1 | 0.582052414 | 6.023794125 | 6.099580705 | 2.01E-09 | 1.06E-08 |
| FGFR1 | 0.540810701 | 7.508826859 | 6.098756815 | 2.02E-09 | 1.06E-08 |
| IFNB1 | -0.394462015 | 0.61315362 | -6.091749888 | 2.10E-09 | 1.10E-08 |
| PSEN1 | 0.175020429 | 7.78454623 | 6.079106472 | 2.26E-09 | 1.18E-08 |
| CUL1 | -0.226692292 | 8.300578672 | -6.06827154 | 2.41E-09 | 1.25E-08 |
| PDK1 | -0.392925278 | 7.237526776 | -6.064356822 | 2.47E-09 | 1.28E-08 |
| CHKA | 0.388560247 | 7.218608434 | 6.051181535 | 2.66E-09 | 1.37E-08 |
| CDK1 | -0.530314422 | 7.07837619 | -6.048363306 | 2.71E-09 | 1.39E-08 |
| MAOA | 0.729282911 | 4.930924989 | 6.038227205 | 2.87E-09 | 1.47E-08 |
| NOX4 | 0.487189571 | 5.370799202 | 6.033609126 | 2.95E-09 | 1.50E-08 |
| MYO9A | 0.252341179 | 7.694758489 | 6.012700921 | 3.33E-09 | 1.69E-08 |
| NOL3 | 0.394422547 | 6.263263459 | 6.009886677 | 3.38E-09 | 1.71E-08 |
| TAT | 1.109116144 | 2.584427256 | 6.009260652 | 3.39E-09 | 1.71E-08 |
| EPAS1 | 0.421530395 | 8.453685929 | 5.976463177 | 4.10E-09 | 2.06E-08 |
| ACSL4 | -0.362636542 | 7.606994748 | -5.969991475 | 4.26E-09 | 2.13E-08 |
| RTN4 | 0.214942568 | 9.331277748 | 5.966743105 | 4.34E-09 | 2.16E-08 |
| FCGR3B | -0.659396871 | 3.095052937 | -5.961974419 | 4.46E-09 | 2.21E-08 |
| TEK | 0.362360842 | 5.404041906 | 5.955088931 | 4.64E-09 | 2.29E-08 |
| GRB2 | -0.217321621 | 8.709781902 | -5.951595881 | 4.73E-09 | 2.33E-08 |
| RAD51 | -0.439059963 | 5.217053662 | -5.931628229 | 5.31E-09 | 2.60E-08 |
| TGM2 | -0.470643355 | 7.471513416 | -5.924383015 | 5.53E-09 | 2.70E-08 |
| PKD1 | 0.31724078 | 7.482804512 | 5.918111045 | 5.73E-09 | 2.79E-08 |
| NGF | 0.613350034 | 1.888339813 | 5.917015855 | 5.77E-09 | 2.80E-08 |
| CCR6 | -0.466586763 | 1.631026272 | -5.901863032 | 6.29E-09 | 3.04E-08 |
| MMP1 | -1.208355693 | 6.091830726 | -5.894657703 | 6.55E-09 | 3.14E-08 |
| PIK3R1 | 0.456530547 | 8.632105855 | 5.894600795 | 6.56E-09 | 3.14E-08 |
| KCNE1 | -0.390561524 | 3.341572797 | -5.885184992 | 6.92E-09 | 3.30E-08 |
| ADA | -0.326906115 | 5.426008913 | -5.87720936 | 7.24E-09 | 3.44E-08 |
| CASQ2 | 0.617349866 | 2.166126162 | 5.867503443 | 7.65E-09 | 3.62E-08 |
| HSP90B1 | -0.249875822 | 11.32018099 | -5.857675229 | 8.09E-09 | 3.82E-08 |
| FRZB | 0.784064254 | 5.208610212 | 5.854547703 | 8.23E-09 | 3.87E-08 |
| CSK | -0.256925973 | 7.454455264 | -5.84344556 | 8.77E-09 | 4.11E-08 |
| ECE1 | 0.33935405 | 8.523880469 | 5.829936223 | 9.46E-09 | 4.42E-08 |
| MKI67 | -0.510777535 | 8.668505148 | -5.825194448 | 9.72E-09 | 4.52E-08 |
| MAPK3 | 0.238751114 | 6.914960793 | 5.82065852 | 9.97E-09 | 4.62E-08 |
| TNIP1 | -0.235765543 | 8.1613559 | -5.80220685 | 1.11E-08 | 5.11E-08 |
| EIF2B4 | 0.220360901 | 6.828238741 | 5.793625806 | 1.16E-08 | 5.34E-08 |
| SDHB | -0.22470735 | 7.542399101 | -5.792732796 | 1.17E-08 | 5.35E-08 |
| PNPT1 | -0.283711479 | 7.386148695 | -5.780517569 | 1.25E-08 | 5.68E-08 |
| IL1RN | -0.632959589 | 4.794569217 | -5.780128947 | 1.25E-08 | 5.68E-08 |
| VIP | 0.36658386 | 1.24282245 | 5.780022617 | 1.25E-08 | 5.68E-08 |
| C9orf72 | -0.325121031 | 6.173325824 | -5.77650584 | 1.28E-08 | 5.77E-08 |
| SP1 | 0.193019502 | 8.744680088 | 5.757892567 | 1.42E-08 | 6.38E-08 |
| DLG4 | 0.294139985 | 4.928048027 | 5.703041143 | 1.92E-08 | 8.63E-08 |
| BRCA2 | -0.344735565 | 6.628150662 | -5.694501112 | 2.02E-08 | 9.02E-08 |
| COX5A | -0.269029285 | 7.765023107 | -5.675235326 | 2.24E-08 | 1.00E-07 |
| AGTR1 | 0.568385629 | 2.817807493 | 5.666886379 | 2.35E-08 | 1.04E-07 |
| FTL | -0.374268704 | 12.98513349 | -5.661669906 | 2.42E-08 | 1.07E-07 |
| PRKAA2 | 0.755094622 | 5.079704265 | 5.632850451 | 2.83E-08 | 1.25E-07 |
| EGR1 | 0.585406264 | 7.692342217 | 5.60638681 | 3.27E-08 | 1.44E-07 |
| MYH6 | -0.385837439 | 0.760501101 | -5.596286102 | 3.46E-08 | 1.51E-07 |
| PON2 | 0.309719638 | 8.013624184 | 5.595450437 | 3.47E-08 | 1.52E-07 |
| YAP1 | 0.295018255 | 8.428819228 | 5.590558506 | 3.57E-08 | 1.55E-07 |
| PDGFRL | 0.474070763 | 5.658133902 | 5.590445785 | 3.57E-08 | 1.55E-07 |
| GYG1 | -0.233665443 | 7.264326911 | -5.583166928 | 3.72E-08 | 1.60E-07 |
| CD28 | -0.444702304 | 4.968282687 | -5.576868822 | 3.84E-08 | 1.66E-07 |
| NEK1 | 0.229136866 | 6.461031458 | 5.545026849 | 4.57E-08 | 1.96E-07 |
| SLC7A11 | -0.48326131 | 5.340597242 | -5.536751137 | 4.78E-08 | 2.04E-07 |
| STK4 | -0.233627431 | 8.001415204 | -5.496773154 | 5.93E-08 | 2.53E-07 |
| DNAH8 | -0.503172063 | 3.394929695 | -5.4906268 | 6.13E-08 | 2.60E-07 |
| ALOX15 | 0.870601507 | 2.531558362 | 5.485786586 | 6.29E-08 | 2.66E-07 |
| CDKN3 | -0.453656266 | 5.373689215 | -5.482863306 | 6.39E-08 | 2.70E-07 |
| SDC1 | 0.547684914 | 10.06227287 | 5.477522565 | 6.57E-08 | 2.76E-07 |
| SLC25A4 | 0.323845567 | 5.876608323 | 5.452830864 | 7.50E-08 | 3.14E-07 |
| SLC1A1 | 0.616720616 | 5.022901751 | 5.430548587 | 8.45E-08 | 3.53E-07 |
| LOX | 0.429998152 | 7.338231752 | 5.407018574 | 9.57E-08 | 3.98E-07 |
| SESN1 | 0.306343437 | 6.521019908 | 5.393180548 | 1.03E-07 | 4.27E-07 |
| TFRC | -0.387134373 | 9.170364305 | -5.390075483 | 1.05E-07 | 4.33E-07 |
| GLT8D1 | 0.190761096 | 7.114259567 | 5.372383039 | 1.15E-07 | 4.73E-07 |
| ODC1 | -0.382099637 | 7.87412088 | -5.372144812 | 1.15E-07 | 4.73E-07 |
| HRH1 | 0.324675391 | 4.91487269 | 5.357743388 | 1.24E-07 | 5.08E-07 |
| GDF15 | 0.73966673 | 3.405161434 | 5.335371333 | 1.40E-07 | 5.70E-07 |
| HSPA14 | -0.286339423 | 7.06101507 | -5.332192602 | 1.42E-07 | 5.78E-07 |
| DLST | 0.148408976 | 7.9027668 | 5.330977369 | 1.43E-07 | 5.80E-07 |
| MT-ND1 | 0.525221886 | 11.43055181 | 5.320898631 | 1.51E-07 | 6.09E-07 |
| MCL1 | -0.251557981 | 10.79083917 | -5.302004505 | 1.66E-07 | 6.70E-07 |
| TYR | 0.402365704 | 1.533951459 | 5.269771969 | 1.96E-07 | 7.90E-07 |
| CXCL1 | -0.746292366 | 4.134532851 | -5.266759458 | 2.00E-07 | 8.00E-07 |
| YBX1 | -0.315810733 | 10.64177862 | -5.256414144 | 2.11E-07 | 8.41E-07 |
| PRDX4 | -0.301705463 | 8.34096663 | -5.249355438 | 2.18E-07 | 8.69E-07 |
| TNFRSF10A | -0.277110998 | 5.982822449 | -5.214508869 | 2.61E-07 | 1.04E-06 |
| PLAUR | -0.382401911 | 7.024711342 | -5.199396304 | 2.82E-07 | 1.12E-06 |
| CPT1A | 0.328365874 | 7.679972407 | 5.198712957 | 2.83E-07 | 1.12E-06 |
| SERPINA1 | -0.562242379 | 6.139176813 | -5.186770999 | 3.01E-07 | 1.18E-06 |
| MBP | -0.32866423 | 6.704047013 | -5.185021052 | 3.04E-07 | 1.19E-06 |
| BLK | -0.770446848 | 3.314117114 | -5.178794359 | 3.14E-07 | 1.23E-06 |
| SREBF1 | 0.465724986 | 8.261086049 | 5.161368467 | 3.43E-07 | 1.34E-06 |
| CAV1 | 0.450652548 | 7.352367628 | 5.157331297 | 3.50E-07 | 1.36E-06 |
| GGT1 | 0.724975577 | 5.95107788 | 5.156850313 | 3.51E-07 | 1.36E-06 |
| MT-ND4 | 0.395473567 | 12.73637962 | 5.151543516 | 3.61E-07 | 1.39E-06 |
| CFLAR | -0.221745095 | 8.65018589 | -5.101895865 | 4.64E-07 | 1.78E-06 |
| TLR3 | -0.282592657 | 5.050465689 | -5.086591201 | 5.01E-07 | 1.92E-06 |
| ACADL | 0.848777055 | 2.858930455 | 5.082058757 | 5.13E-07 | 1.96E-06 |
| HGF | 0.353902314 | 5.033698371 | 5.058356231 | 5.77E-07 | 2.20E-06 |
| ITGB3 | 0.368633009 | 4.13221726 | 5.052111258 | 5.96E-07 | 2.26E-06 |
| PTPN3 | 0.346872961 | 6.689511244 | 5.051870837 | 5.96E-07 | 2.26E-06 |
| PTPA | 0.237047312 | 8.437071496 | 5.051584505 | 5.97E-07 | 2.26E-06 |
| GLS | -0.341394153 | 8.795135634 | -5.049785935 | 6.03E-07 | 2.27E-06 |
| IL2 | -0.290066571 | 0.634774917 | -5.032348999 | 6.57E-07 | 2.47E-06 |
| MAPK8IP1 | 0.398382966 | 4.576459677 | 5.015705338 | 7.14E-07 | 2.68E-06 |
| DHCR24 | 0.642946122 | 9.373589123 | 4.997966185 | 7.80E-07 | 2.91E-06 |
| COMT | 0.266264773 | 7.850801715 | 4.982869494 | 8.41E-07 | 3.13E-06 |
| EDNRB | 0.452700101 | 5.263586524 | 4.979514288 | 8.55E-07 | 3.17E-06 |
| MYD88 | -0.22024196 | 7.431836159 | -4.969891283 | 8.96E-07 | 3.32E-06 |
| SIAH1 | 0.190786439 | 5.827926655 | 4.961791032 | 9.33E-07 | 3.44E-06 |
| G6PD | 0.418992161 | 7.327330492 | 4.952204583 | 9.78E-07 | 3.60E-06 |
| ETFB | 0.375708874 | 5.47331804 | 4.941705519 | 1.03E-06 | 3.78E-06 |
| NRAS | -0.340858743 | 7.943789172 | -4.933785563 | 1.07E-06 | 3.92E-06 |
| DDAH1 | 0.362102363 | 7.106525438 | 4.914201739 | 1.18E-06 | 4.30E-06 |
| CD55 | 0.311447654 | 7.931346857 | 4.912887713 | 1.19E-06 | 4.31E-06 |
| DYNC1H1 | 0.189228637 | 10.64125823 | 4.907081405 | 1.22E-06 | 4.43E-06 |
| ENO1 | -0.283916716 | 11.40764593 | -4.888132347 | 1.34E-06 | 4.84E-06 |
| ATXN1 | 0.237547115 | 7.707639823 | 4.84030739 | 1.69E-06 | 6.09E-06 |
| GPX2 | 0.378683643 | 4.15480824 | 4.837691656 | 1.71E-06 | 6.15E-06 |
| JUN | 0.295773737 | 8.925321955 | 4.832659883 | 1.75E-06 | 6.28E-06 |
| BCL6 | 0.306068492 | 7.532401877 | 4.818617148 | 1.87E-06 | 6.70E-06 |
| CHEK1 | -0.364399301 | 6.155887538 | -4.811912778 | 1.93E-06 | 6.90E-06 |
| DBH | -0.403622219 | 2.076558982 | -4.806579912 | 1.98E-06 | 7.06E-06 |
| TLR4 | -0.313146207 | 6.790521643 | -4.804320762 | 2.01E-06 | 7.12E-06 |
| AKR1B1 | -0.312664436 | 6.90724822 | -4.8012496 | 2.04E-06 | 7.20E-06 |
| DAXX | -0.500605155 | 3.180722225 | -4.799814878 | 2.05E-06 | 7.23E-06 |
| TRPV1 | 0.188713133 | 4.511255584 | 4.797784793 | 2.07E-06 | 7.28E-06 |
| BAX | -0.204467688 | 6.867060701 | -4.781218182 | 2.24E-06 | 7.86E-06 |
| HPRT1 | -0.325790938 | 6.677943342 | -4.7776227 | 2.28E-06 | 7.97E-06 |
| NFKB1 | -0.188874211 | 7.779074718 | -4.773533204 | 2.32E-06 | 8.11E-06 |
| PLCG1 | 0.226655766 | 8.141360115 | 4.75999107 | 2.48E-06 | 8.62E-06 |
| MUC1 | 0.750785822 | 7.24166214 | 4.754664645 | 2.54E-06 | 8.82E-06 |
| PARP1 | -0.224550691 | 9.336455608 | -4.741028785 | 2.71E-06 | 9.39E-06 |
| MAPKAPK3 | -0.186099013 | 7.080408092 | -4.721618441 | 2.97E-06 | 1.03E-05 |
| VCL | 0.255003349 | 9.108397657 | 4.697842459 | 3.33E-06 | 1.14E-05 |
| MYH7 | -0.350792154 | 0.57969755 | -4.697496061 | 3.33E-06 | 1.14E-05 |
| MSR1 | -0.326270968 | 7.577547192 | -4.697270048 | 3.34E-06 | 1.14E-05 |
| SNTA1 | 0.319591653 | 5.666916494 | 4.694239132 | 3.38E-06 | 1.15E-05 |
| MT-CO1 | 0.256221487 | 14.28846039 | 4.689793021 | 3.45E-06 | 1.18E-05 |
| TGFBR1 | 0.254645929 | 8.31537159 | 4.676010383 | 3.69E-06 | 1.25E-05 |
| C1QBP | -0.255041697 | 7.981374365 | -4.675160795 | 3.70E-06 | 1.25E-05 |
| VEGFC | 0.291164589 | 4.97906677 | 4.672433188 | 3.75E-06 | 1.27E-05 |
| CYP27A1 | -0.377184195 | 7.372783352 | -4.658094494 | 4.01E-06 | 1.35E-05 |
| FMR1 | -0.205769467 | 7.752355288 | -4.64605345 | 4.24E-06 | 1.42E-05 |
| ELN | 0.727492377 | 6.839228341 | 4.643338538 | 4.29E-06 | 1.44E-05 |
| TXN2 | 0.199504425 | 7.333335401 | 4.63963345 | 4.37E-06 | 1.46E-05 |
| OLR1 | -0.492220167 | 6.17541237 | -4.630506769 | 4.56E-06 | 1.52E-05 |
| SLC1A3 | -0.335306081 | 6.866822818 | -4.614589926 | 4.91E-06 | 1.63E-05 |
| ETFA | 0.277885792 | 7.750095236 | 4.606274382 | 5.10E-06 | 1.69E-05 |
| ANG | 0.350803177 | 3.75677553 | 4.601952431 | 5.20E-06 | 1.72E-05 |
| ABCC3 | 0.418590378 | 6.172676875 | 4.601593229 | 5.21E-06 | 1.72E-05 |
| GSR | 0.325095176 | 7.721900028 | 4.59535575 | 5.36E-06 | 1.76E-05 |
| GSTM5 | 0.695717271 | 2.442182274 | 4.584937916 | 5.63E-06 | 1.85E-05 |
| GRIN2A | 0.587433562 | 3.146418224 | 4.581335967 | 5.72E-06 | 1.87E-05 |
| UCHL1 | -0.695158478 | 5.853812147 | -4.557297575 | 6.39E-06 | 2.09E-05 |
| KRT18 | 0.48904929 | 9.040683247 | 4.554110141 | 6.48E-06 | 2.11E-05 |
| NR4A2 | 0.361796788 | 5.310946115 | 4.547320798 | 6.69E-06 | 2.17E-05 |
| HFE | 0.260691474 | 5.612881352 | 4.543049942 | 6.82E-06 | 2.21E-05 |
| PENK | 0.528813244 | 1.047126365 | 4.540550234 | 6.90E-06 | 2.23E-05 |
| NDUFS1 | 0.163532391 | 8.094347609 | 4.531150659 | 7.20E-06 | 2.32E-05 |
| HLA-DRB1 | -1.158876678 | 6.840515702 | -4.526432562 | 7.36E-06 | 2.37E-05 |
| INSR | 0.269670012 | 8.400784578 | 4.497762744 | 8.38E-06 | 2.69E-05 |
| CALR | -0.182488752 | 11.36867705 | -4.496714243 | 8.42E-06 | 2.69E-05 |
| RUNX2 | 0.290116277 | 6.43039768 | 4.461595526 | 9.87E-06 | 3.15E-05 |
| IDH1 | 0.314680466 | 8.441399022 | 4.440385531 | 1.09E-05 | 3.46E-05 |
| PSIP1 | -0.324083131 | 7.823348789 | -4.439493287 | 1.09E-05 | 3.46E-05 |
| THBS1 | 0.412362419 | 10.65728873 | 4.439184406 | 1.09E-05 | 3.46E-05 |
| TUBA1B | -0.203196465 | 9.270200884 | -4.435676422 | 1.11E-05 | 3.50E-05 |
| DUSP19 | 0.223005432 | 3.908205055 | 4.430960205 | 1.13E-05 | 3.57E-05 |
| OXTR | 0.482823052 | 4.437984088 | 4.420454517 | 1.19E-05 | 3.73E-05 |
| SHC1 | 0.179232206 | 8.380369129 | 4.416775192 | 1.21E-05 | 3.78E-05 |
| HSPA6 | -0.432566913 | 5.01210494 | -4.416169355 | 1.21E-05 | 3.79E-05 |
| ADCY10 | 0.272258796 | 3.18345375 | 4.411659063 | 1.23E-05 | 3.85E-05 |
| GCLC | 0.217012488 | 6.967952644 | 4.396498375 | 1.32E-05 | 4.11E-05 |
| IL1R1 | 0.324548787 | 7.844571437 | 4.383907322 | 1.40E-05 | 4.34E-05 |
| DNMT1 | -0.238206039 | 8.417873395 | -4.380111415 | 1.42E-05 | 4.40E-05 |
| PRKCG | -0.315053372 | 0.943105961 | -4.375209368 | 1.45E-05 | 4.49E-05 |
| C5AR1 | -0.316814421 | 5.581349417 | -4.367367649 | 1.50E-05 | 4.63E-05 |
| GSS | 0.231246798 | 7.285597684 | 4.360880092 | 1.55E-05 | 4.75E-05 |
| MT-ND3 | 0.331265268 | 11.571169 | 4.360751145 | 1.55E-05 | 4.75E-05 |
| C3 | -0.504311346 | 10.28777277 | -4.35335206 | 1.60E-05 | 4.89E-05 |
| CACNA2D1 | 0.306773529 | 5.492570018 | 4.351781998 | 1.61E-05 | 4.92E-05 |
| TYRP1 | 0.522845669 | 1.467368009 | 4.339829255 | 1.70E-05 | 5.17E-05 |
| MAPK9 | 0.189273772 | 7.019021332 | 4.338940322 | 1.70E-05 | 5.18E-05 |
| BMP6 | -0.411700323 | 4.510159835 | -4.334602813 | 1.74E-05 | 5.26E-05 |
| OSGIN1 | 0.397824736 | 3.902662675 | 4.33287739 | 1.75E-05 | 5.28E-05 |
| VAPB | 0.191184202 | 7.688929507 | 4.332740526 | 1.75E-05 | 5.28E-05 |
| KEAP1 | -0.201978557 | 7.471840388 | -4.324464119 | 1.82E-05 | 5.46E-05 |
| SCN4A | 0.432381447 | 2.542973082 | 4.31980898 | 1.85E-05 | 5.56E-05 |
| TPM1 | 0.303643531 | 9.449463959 | 4.316269574 | 1.88E-05 | 5.64E-05 |
| GSTM2 | 0.359876856 | 4.699480128 | 4.30401955 | 1.99E-05 | 5.93E-05 |
| SLC25A1 | 0.244638546 | 7.540021576 | 4.303686274 | 1.99E-05 | 5.93E-05 |
| GSTA4 | 0.360355489 | 6.322478133 | 4.278368889 | 2.22E-05 | 6.60E-05 |
| NDUFA10 | 0.150723952 | 7.849953893 | 4.265802325 | 2.35E-05 | 6.92E-05 |
| GADD45B | 0.298316616 | 7.432416267 | 4.265793862 | 2.35E-05 | 6.92E-05 |
| CTTN | 0.229021508 | 9.375842169 | 4.265682618 | 2.35E-05 | 6.92E-05 |
| KNG1 | -0.26577499 | 0.86762214 | -4.265528373 | 2.35E-05 | 6.92E-05 |
| CD46 | 0.228230755 | 9.544890004 | 4.259564639 | 2.41E-05 | 7.08E-05 |
| FKBP1B | 0.306718242 | 2.39858553 | 4.24275532 | 2.59E-05 | 7.60E-05 |
| CAMK2G | 0.177179137 | 6.979804061 | 4.238504165 | 2.64E-05 | 7.72E-05 |
| GFM2 | 0.177515748 | 6.529678382 | 4.232592838 | 2.71E-05 | 7.90E-05 |
| FMO3 | 0.509231952 | 4.796912826 | 4.194538238 | 3.19E-05 | 9.28E-05 |
| EGFR | 0.472731287 | 7.805686995 | 4.191758159 | 3.23E-05 | 9.37E-05 |
| CASP4 | -0.220128405 | 7.213924447 | -4.189970708 | 3.25E-05 | 9.42E-05 |
| AQP1 | 0.448430619 | 7.567623685 | 4.180707425 | 3.38E-05 | 9.78E-05 |
| TCF7L2 | 0.250658283 | 6.989501838 | 4.177954075 | 3.42E-05 | 9.87E-05 |
| GJA1 | 0.381804443 | 8.210450996 | 4.177193472 | 3.43E-05 | 9.88E-05 |
